# Oilseed Processing By-Products for Biodegradable Food Packaging: Chemical Complementarity and Interfacial Organization

**DOI:** 10.3390/molecules31214021

**Published:** 2026-11-08

**Authors:** Joanna Igielska-Kalwat, Magdalena Rudzińska

**Affiliations:** Department of Food Technology of Plant Origin, Faculty of Food Science and Nutrition, Poznań University of Life Sciences, Wojska Polskiego 28, 60-637 Poznań, Poland

**Keywords:** oilseed side streams, oil cakes, soapstock, phospholipids, biodegradable food packaging, interfacial interactions, barrier properties, active packaging, food-contact materials, lipid-rich foods

## Abstract

Biodegradable food-packaging materials can be developed from multiple agricultural and food-processing co-products, including by-products generated during oilseed processing. These oilseed-derived side streams contain proteins, polysaccharides, fibers, residual lipids, free fatty acids, phospholipids, sterols, tocopherols, and phenolic compounds, which may provide complementary functions within multicomponent packaging systems. This review critically examines oil cakes and meals as sources of structural components, residual oils and fractions recovered from soapstock as hydrophobic phases, and lecithin and other phospholipid-rich fractions as regulators of interfacial organization. Particular attention is given to the relationships between chemical composition, phase organization, material microstructure, and the resulting mechanical, barrier, and active properties. The review also discusses processing effects, oxidative stability, migration, food-contact safety, and functional validation in lipid-rich foods. The distinctive contribution is an evidence-informed, function-oriented synthesis that links the chemical identity of recovered fractions to interfacial organization, material performance, and verified food protection rather than treating side streams as interchangeable feedstocks. Further progress in this field will require improved compositional standardization, control of interfacial organization, validation under realistic processing and storage conditions, and direct demonstration of food protection.

## 1. Introduction

Packaging is an integral element of food protection systems, and its function extends beyond the physical separation of the product from the surrounding environment. Modern packaging materials are expected to provide adequate mechanical strength, control oxygen and water vapor transfer, and limit the effects of light and other factors that accelerate product deterioration. At the same time, development in the packaging sector is increasingly directed toward reducing the use of petrochemical materials and increasing the contribution of renewable, biodegradable resources, including those derived from food-industry by-products [1,2,3].

Natural biopolymers, particularly proteins and polysaccharides, form the basis of many biodegradable film-forming materials, although their hydrophilic character may limit barrier performance under conditions of elevated humidity [3,4]. Lipid components fulfill a different function. They may reduce the affinity of the material for water and modify water vapor transport by altering system polarity and phase organization. Their effectiveness, however, depends not only on concentration but also on physical state, degree of dispersion, compatibility with the matrix, and interfacial stability [3,5].

Oilseed processing is particularly relevant in this context because a single technological chain generates side streams with markedly different chemical compositions. Oil cakes and meals remaining after pressing or extraction contain proteins, polysaccharides, fiber, phenolic compounds, and variable amounts of residual oil. During purification and refining, phospholipid-rich fractions, soapstock, and distillates containing free fatty acids, acylglycerols, sterols, tocopherols, and other minor constituents are generated [6,7,8]. These chemically distinct fractions may therefore contribute different structural, hydrophobic, interfacial, or active functions to packaging materials.

Other agricultural and food-industry co-products are also being investigated for biodegradable packaging, including residues from cereal and pulse processing and from fruit and vegetable processing, which may contribute film-forming biopolymers, reinforcing components, or compounds with potential active functionality [1,4,5]. Accordingly, the present review does not suggest that oilseed-derived fractions are the predominant feedstocks for such materials or that their utilization is inherently more sustainable. Its narrower focus is justified by the chemical complementarity of solid and lipid-rich streams generated during successive oilseed-processing operations, which allows structural, hydrophobic, and interfacial functions to be examined within a common materials-design framework [1,6,7,8]. Any technological or environmental advantage of combining these fractions must nevertheless be demonstrated through appropriate processing, material characterization, food-protection testing, and, where relevant, environmental assessment.

Studies using canola protein isolate have produced films by laboratory-scale solution casting [9,10]. Their measured properties depended on protein and glycerol concentrations [9] and on the incorporation of sodium dodecyl sulfate or stearic acid [10]. Direct incorporation of whole oil cakes offers a different route, although it usually sacrifices compositional control [1,11]. A rapeseed oil-cake film has also been tested directly as a package for sunflower oil, providing food-level evidence beyond film characterization [12]. Refining-derived by-products have received less comparable investigation: films have been made from soapstock fractions [13], and soapstock-derived coatings have reduced moisture loss from bell peppers [14]. However, these studies do not yet establish the performance of deliberately combined oilcake–soapstock–lecithin formulations.

Phospholipid-rich fractions represent a possible means of regulating the hydrophilic–lipid interface, but their measured effect depends on the matrix, lipid loading, and post-forming treatment, as shown in cast chitosan and alginate emulsion films [15]. This dependence must be distinguished from proof of superior performance of unrefined oilseed-derived lecithin fractions.

Existing reviews provide complementary, but partly overlapping, perspectives. Petraru and Amariei [2] concentrated on oilcake-derived edible packaging, including protein-rich films and the potential incorporation of whole oilcakes. Dissanayake et al. [1] surveyed a broader range of pulse- and oilseed-industry coproducts, their biopolymers, additives, processing technologies, and packaging applications. The review by Devi et al. [3] examined lipid-containing biopolymer films and coatings across different feedstocks. These contributions already establish the relevance of coproduct utilization and of composition–property relationships; neither concept is claimed as new here. The distinct contribution of the present review is to connect chemically different streams generated within oilseed processing—structural cake/meal fractions, lipid-rich refining fractions, and phospholipid-rich interfacial fractions—within a single, critical, evidence-informed structure–function synthesis. It examines when the proposed roles are supported by studies of defined material systems and when they remain hypotheses requiring direct tests of processing stability, food protection, and safety [1,2,3,6,7,8,12,15].

The evaluation of such materials should extend beyond their physical properties to include their actual ability to protect food. This is particularly important for lipid-rich products, in which oxygen availability, light exposure, and storage temperature directly influence oxidation. Packaging type has been shown to affect the stability of vegetable oils, including peroxide value, tocopherol and sterol contents, formation of phytosterol oxidation products, and changes in triacylglycerol composition during storage [16]. These observations support the use of food-level chemical responses as a complementary measure of packaging performance rather than relying solely on the properties of the material itself.

The aim of this review is to critically evaluate how chemically characterized oilseed-processing by-products and their recovered fractions can fulfill structural, hydrophobic, interfacial, and active roles in biodegradable food packaging, while distinguishing experimentally demonstrated material properties from validated food-protection outcomes.

## 2. Scope and Concept of the Review

**Figure 1:**
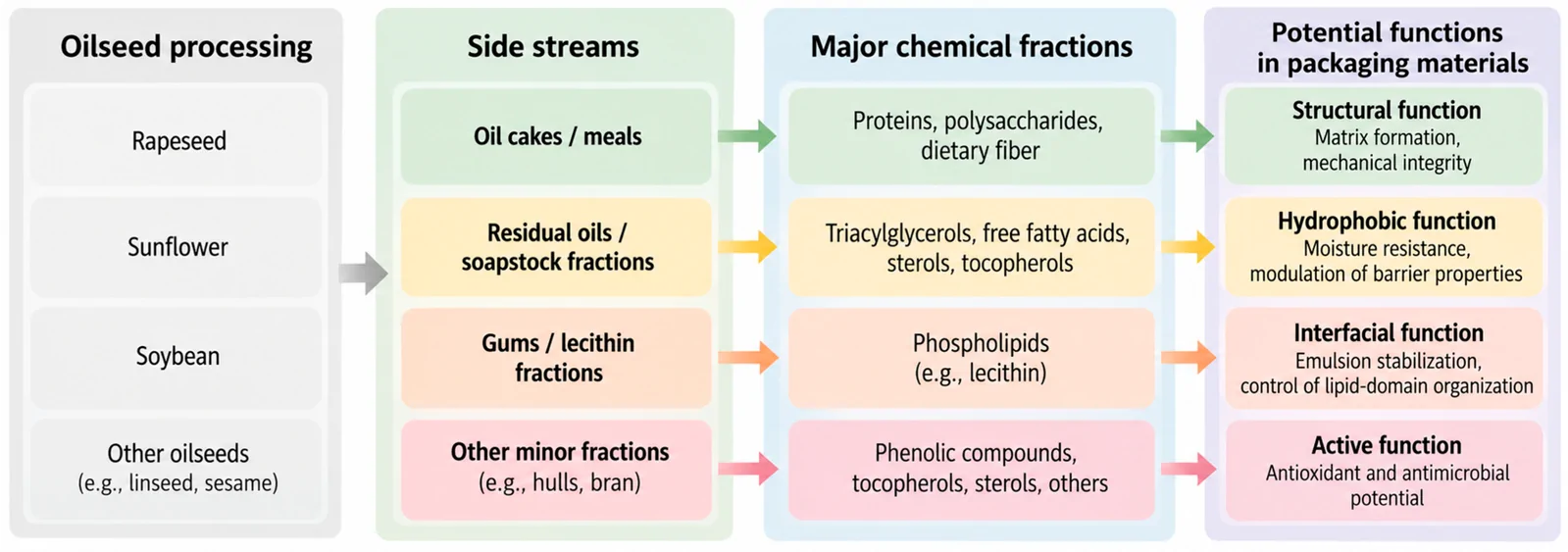
Conceptual scheme of oilseed-processing side streams and their potential structural, hydrophobic, interfacial, and active roles in multicomponent biodegradable food-packaging materials.

The review is organized around two complementary analytical axes. The first distinguishes the origin of an oilseed-processing side stream from the function assigned to its recovered constituents—structural, hydrophobic, interfacial, or potentially active. These categories are developed in Section 3 and applied throughout the subsequent analysis (Figure 1). As individual fractions may contribute to more than one function, their classification identifies candidate roles within specific formulations rather than presuming improved material performance [1,2,3,6,7,8].

The second axis concerns the type and strength of experimental evidence. We distinguish (i) chemical characterization and plausible functionality, (ii) direct characterization of films or coatings, (iii) functional tests in simplified model systems, and (iv) evidence of improved preservation of a packaged food under defined storage conditions. Findings obtained using purified model ingredients or non-oilseed systems can provide mechanistic insights but are not treated as direct validation of compositionally variable industrial side streams [11,12,14,15,17,18,19].

Comparisons prioritize within-study contrasts and consider, where reported, composition, film thickness, plasticizer loading, preparation method, conditioning conditions, and test protocols. Numerical differences across studies are interpreted cautiously when relative-humidity gradients, temperature, permeability units, or mechanical testing methods are not aligned. For each functional role, the analysis considers the observed response, plausible explanations, performance trade-offs, and remaining confounders. These criteria are also applied to whole-stream utilization and fractionation, including the practical requirements of additional processing [1,2,9,11,15].

Section 3 establishes the functional classification of recovered fractions. Section 4 analyzes structural components, Section 5 lipid-rich fractions, and Section 6 phospholipid-mediated interfacial regulation. Section 7 integrates structure–property findings, including active functionality; Section 8 considers processing strategies; Section 9 evaluates food-protection performance, stability, safety, and end-of-life; and Section 10 translates unresolved limitations into testable research priorities.

### Literature Search and Selection Strategy

This article is a critical narrative review, not a systematic review or meta-analysis. For this revision, the existing bibliography was re-evaluated through targeted searches and verification of publisher records, PubMed-indexed records where available, and EUR-Lex for European food-contact legislation (source check: 3 November 2026). Search terms were combined around the feedstock and its function, including ‘oilseed’, ‘oil cake’, ‘oilseed meal’, ‘rapeseed’, ‘canola’, ‘sunflower’, ‘soapstock’, ‘lecithin’, and ‘phospholipid’, with ‘biodegradable film’, ‘food packaging’, ‘coating’, ‘interfacial organization’, ‘barrier properties’, ‘oxidation’, ‘migration’, and ‘food preservation’. The references considered include foundational studies from 1999 and relevant work published through 2026. The original literature-identification process was not recorded as a reproducible database search, and no complete screening log or study-selection counts are available; these have therefore not been reconstructed retrospectively.

Evidence selection was based on relevance to the chemistry, recovery route and intended structural, hydrophobic, interfacial or active function of oilseed-derived fractions. Priority was given to experimental publications that identify the studied feedstock, formulation and processing conditions, and report mechanical, transport, microstructural, active or food-protection outcomes. Foundational reviews and non-oilseed biopolymer–lipid experiments were included only to contextualize mechanisms and limits of transferability; regulatory and standardized-testing sources were used for safety and end-of-life discussions. Comparisons were made primarily within each study and interpreted in relation to film thickness, formulation basis, humidity, measurement units, storage conditions and the presence or absence of a packaged-food trial. Because the formulations and test protocols are heterogeneous, no statistical meta-analysis was performed.

## 3. Oilseed Processing By-Products as Sources of Chemically Complementary Fractions

### 3.1. Generation and Chemical Diversity of Oilseed-Processing Side Streams

Oilseed processing produces chemically distinct side streams whose potential use in packaging reflects both seed composition and processing history. Mechanical pressing yields protein- and fiber-containing cake with residual oil, while further solvent extraction generally lowers residual lipid content in the resulting meal [1,2,20]. Degumming yields phospholipid-rich gums, alkaline neutralization produces soapstock containing fatty acid soaps, free fatty acids, acylglycerols and water, and deodorization generates distillates containing fatty acids and minor lipid constituents [6,7,8]. These streams should not be regarded as interchangeable feedstocks: a cake obtained by pressing differs from an extracted meal even when derived from the same oilseed, and the chemical profile of refining streams depends on the operation used. This technological origin provides a basis for describing potential structural, hydrophobic, interfacial and active functions (Figure 1), but does not by itself predict the properties of a finished film or coating. That prediction requires compositional specifications and formulation-level evidence.

The botanical origin of a recovered fraction must be considered together with its processing history. Rapeseed and canola provide particularly instructive examples: whole rapeseed oil cake has been evaluated as a packaging material for protecting sunflower oil during storage [12], whereas canola protein isolates have been investigated as more compositionally defined film-forming matrices [9,10], and canola meal has been used in reinforced nanocomposite films [21]. Rapeseed soapstock has also been studied as a feedstock for acid-oil recovery [22]. These studies concern different fractions and different levels of functional evidence. In particular, performance measured for an isolated protein film cannot be directly extrapolated to an unfractionated oil cake, and successful acid-oil recovery does not by itself demonstrate the suitability of the recovered fraction for food-contact packaging.

The diversity of recovery routes is similarly important for other oilseeds. Sunflower oil cake has been incorporated into edible-membrane formulations [11], whereas sunflower seed proteins have been combined with locust bean gum in polymeric films [23], and sunflower protein isolate has been investigated in film extrusion [24]. Cottonseed protein has been used in bilayer packaging films [25], while cottonseed and safflower soapstocks have been investigated as starting materials for soapstock-derived films [13]. These examples demonstrate that even when fractions originate from the same class of oil crops, the resulting materials differ in chemical composition, degree of fractionation, matrix architecture, and processing route. They should therefore be compared according to the recovered fraction and demonstrated material function, rather than by oilseed name alone [1,2].

The distinction between botanical source and processing stage is especially relevant to refining by-products. Phospholipid-rich gums are obtained during degumming, soapstock is associated with alkaline neutralization, and deodorizer distillates originate from the deodorization stage [6,7,8]. Their occurrence and composition therefore depend on the refining process and cannot be assumed to be identical for every oilseed or oil-production route. Although soybean, flaxseed, hemp, and other oilseeds represent additional potential sources of structural or lipid-rich fractions [1,2], their suitability for a specific packaging function requires direct feedstock-specific evidence. For meaningful cross-study comparisons, the oilseed species, recovery and refining route, degree of fractionation, residual-lipid composition and oxidative state, and level of packaging validation should be reported wherever available.

### 3.2. Compositional Variability as a Material-Design Parameter

Variability among oilseed side streams is an experimental confounder as well as a potential resource. The contents of protein, fiber, residual oil and phenolics depend on cultivar, seed conditioning and oil-recovery route [1,2]; soapstock composition additionally depends on degumming and neutralization conditions [6,7]. For meaningful comparisons, solid feedstocks should be reported on a dry-mass basis, including at least protein, fiber, residual lipid and relevant minor constituents. Lipid-rich fractions require lipid-class and fatty acid profiles, free fatty acid content and oxidative status, with phospholipid, sterol or tocopherol quantification where these components are invoked to explain function [6,7,8]. A nominally identical addition of “oilcake” or “lipid” may therefore represent different effective concentrations of structural or amphiphilic components. Without common sampling, compositional characterization and matched processing conditions, apparent disagreement in film performance may reflect differences between raw materials rather than differences in the proposed design mechanisms. Accordingly, Section 3 defines what needs to be characterized; Section 4, Section 5, Section 6, Section 7 and Section 8 assess whether those descriptors are linked to measurable responses.

### 3.3. Functional Classification of Recovered Fractions

For the design of multicomponent packaging materials, oilseed-processing by-products can be classified not only according to their technological origin but also according to the dominant function their constituents may perform within the material. This classification is necessarily flexible because most recovered fractions are multifunctional and their effects depend on composition, concentration, and interactions with other phases. The first category comprises structural fractions, particularly proteins and polysaccharides originating from oil cakes and meals. Their principal role is to form or reinforce the continuous phase. This ability depends on protein solubility and conformation, intermolecular interactions, and the compatibility of dispersed fibrous components with the surrounding matrix [2,9,10]. The second category includes hydrophobic fractions such as residual oils, cake oils, and lipid fractions recovered from soapstock. Their principal contribution is modification of material polarity and interactions with water. However, increasing lipid content does not necessarily reduce water vapor permeability because the final effect depends on lipid dispersion, domain morphology, and continuity of the polymer phase [3]. The third category comprises interfacial fractions, particularly phospholipids. Lecithin can influence interactions between hydrophilic and lipid phases and modify emulsion structure before film formation. Consequently, phospholipid-containing systems may develop different lipid-domain distributions and material microstructures depending on formulation and processing conditions [15,26]. A fourth category includes fractions containing compounds with potential active functionality. Oilseed-processing by-products may provide phenolic compounds, tocopherols, sterols, and other minor constituents with antioxidant potential [8,17,18,27]. Their presence, however, should not be considered equivalent to active-packaging functionality. Activity must be demonstrated under conditions relevant to the material and, ultimately, to the packaged food. These functional categories are not mutually exclusive. Residual lipids may simultaneously modify hydrophobicity and plasticization, phospholipids may influence both interfacial organization and overall system polarity, and phenolic compounds may affect both antioxidant activity and matrix interactions. Material design should therefore consider the dominant function of each fraction together with its interactions with the remaining phases (Table 1).

### 3.4. From Stream Origin to Controlled Material Function

The four functional categories in Table 1 are applied as evidence-based criteria rather than as mutually exclusive labels or a prescription for a single optimal formulation. Structural contributions are evaluated through matrix formation, tensile behavior, and continuity (Section 4); hydrophobic contributions through surface wettability, water-vapor transport, and chemical stability (Section 5); interfacial contributions through phospholipid-dependent dispersion and the morphology of the dried material (Section 6); and potentially active contributions through identified constituents, incorporation levels, and measured responses, including food-level outcomes where available (Section 7 and Section 9). Section 7 considers trade-offs among these functions, while Section 8 examines processing dependence and Section 9 separates material properties from food preservation. Because individual fractions can serve multiple roles, classification describes their evaluated contribution in a given formulation rather than an intrinsic single function. In the absence of matched formulation studies, chemical complementarity remains a design hypothesis rather than a demonstrated packaging advantage.

## 4. Oil Cakes and Meals as Sources of Structural Components

### 4.1. Whole-Stream and Fraction-Based Approaches to Structural Material Design

Whole-stream use and fraction-based design are not interchangeable routes to the same film. Mechanically pressed cake retains proteins, fiber, residual lipids and minor constituents, whereas extracted meal is generally depleted of residual oil; both may require milling, dispersion, heat treatment or blending with a film-forming polymer before a usable material is obtained [1,2,20]. Protein concentrates or isolates require additional separation and purification but permit more deliberate control of the protein-rich continuous phase and the plasticizer-to-protein ratio [9,10]. Accordingly, their relative merits should be examined in terms of the finished formulation and its processing burden, not simply the nominal cost or composition of the starting by-product. The following studies test distinct formulation strategies, but they are not controlled whole-cake-versus-isolate comparisons.

Petraru and Amariei [11] incorporated 0–0.5 g sunflower oilcake flour into films based on 1.0 g alginate and 0.5 g glycerol. Within that single casting series, tensile strength fell from 27.11 to 8.66 MPa while water vapor permeability (WVP) decreased overall from 1.66 × 10^−4^ to 1.13 × 10^−4^ g mm m^−2^ h^−1^ kPa^−1^. The effects were not uniformly beneficial: at 0.1 g oilcake, WVP increased to 1.98 × 10^−4^ in the same units, even though the highest loading gave the lowest reported WVP. Thickness rose from 0.029 to 0.044 mm and density fell from 1.53 to 1.04 g cm^−3^. Thus, a simple explanation based on greater film density is not supported; furthermore, WVP includes a thickness correction. The results document a non-monotonic transport response accompanied by a marked loss of mechanical strength. Because oilcake was added as an intrinsically mixed filler to an alginate matrix, the study does not isolate the separate effects of its proteins, particles and residual lipids.

Vera et al. [12] obtained a rapeseed-oilcake-based film with a tensile strength of 1.18 MPa, elongation at break of 13.86%, and WVP of 1.43 × 10^−10^ g m^−1^ s^−1^ Pa^−1^. Unlike most material-screening studies, the resulting packages were also assessed with sunflower oil during 60 days of storage at 23 °C and reduced measured oxidation indicators relative to an exposed control. This provides food-level evidence for one whole-stream route, not evidence that whole oilcake is mechanically equivalent or superior to a protein isolate. In particular, comparing these values directly with those of alginate–sunflower-oilcake composites [11] would confound oilseed identity, continuous-phase chemistry, formulation, test conditions and permeability units.

An isolate-based strategy enables more targeted formulation experiments. Chang and Nickerson [9] prepared cast canola-protein-isolate films with 5.0 or 7.5% protein and 30–50% glycerol relative to protein mass. Within these defined matrices, higher glycerol decreased tensile strength and stiffness while increasing extensibility and WVP; a higher protein concentration increased tensile strength but also increased WVP. Greater protein-network contribution therefore did not imply a better moisture barrier. The advantage demonstrated by this study is the ability to distinguish protein and plasticizer effects. It is not a matched performance comparison with intact cake or meal; producing a protein isolate also entails upstream processing that is absent from direct whole-stream incorporation [1,9].

### 4.2. Oilseed Proteins as Components of the Continuous Phase

Protein-rich isolates can form load-bearing continuous phases as molecular interactions develop during casting, solvent removal or thermal processing. Their cohesion is influenced by protein solubility, conformation and the imposed chemical environment [9,10]. This subsection therefore evaluates the protein network itself, rather than repeating the feedstock-selection comparison made in Section 4.1.

The modifier study by Shi and Dumont [10] provides a useful counterexample to the assumption that increasing strength and water resistance must occur together. In canola-protein-isolate films made by solution casting, sodium dodecyl sulfate (SDS) improved mechanical properties but promoted water uptake, whereas stearic-acid co-plasticization reduced water uptake while weakening the films. These effects are attributable to the entire modified formulation, not to protein purification alone. Importantly, water absorption is not interchangeable with WVP: the former characterizes liquid-water uptake or swelling, while the latter measures vapor transport under a specified humidity gradient.

The experiments of Chang and Nickerson [9] and Shi and Dumont [10] together show the interpretive benefit of a chemically narrower starting fraction: changing glycerol, protein loading or a defined additive makes competing mechanical and water-related responses observable. At the same time, they identify different performance trade-offs rather than one optimal isolate formulation. Direct extrapolation to whole oilcake is not justified because proteins coexist there with fibers, residual lipids and other constituents [11].

Recent studies confirm that these relationships remain central even in more advanced oilseed-protein systems. Sunflower seed proteins combined with locust bean gum produced films whose physicochemical, thermal, and mechanical properties depended on polysaccharide concentration, demonstrating that protein–polysaccharide interactions can be deliberately used to modify network organization [23].

Similarly, recent work on canola-protein films showed that chemically tailored graphite oxide markedly altered tensile, thermal, and oxygen-barrier properties through interactions with the protein matrix, further confirming that protein-network functionality depends on both molecular interactions and microstructural organization [28].

For isolated protein films, the relevant measurable advantage is formulation control, not proven superiority over whole-stream materials. Protein-isolation yield and purity, residual lipid concentration, solvent and water requirements, drying conditions and the starting meal composition should all be reported to evaluate the practical benefit of isolation [1,9,10]. In the absence of paired comparisons prepared from the same oilseed lot, neither greater material performance nor a lower overall environmental burden can be inferred for isolates or whole cakes.

### 4.3. Fiber and Polysaccharide Fractions as Reinforcing Components

Fiber and polysaccharide-rich fractions introduce a reinforcing or filler function that is analytically distinct from the film-forming protein network. Their effects depend on particle size, dispersion and adhesion, and may differ between directly incorporated oilcake/meal and a compositionally controlled fiber fraction [1,2]. Their presence should therefore not be treated as evidence of reinforcement without a matched mechanical response.

Uniformly dispersed, well-adhered particles may transfer load and alter transport paths, whereas coarse particles or aggregates may generate defects. However, a change in tensile strength cannot establish fiber-driven reinforcement without information on filler fraction, particle-size distribution and film morphology. The simultaneous presence of residual oil and other compounds in whole cake additionally restricts causal attribution [11].

Oilseed-meal biocomposites reinforced with plant fibers [29] demonstrate that filler addition and compatibility can alter mechanical and physical performance, but plate materials and thin packaging films cannot be evaluated as interchangeable product formats. Their processing and end-use requirements should be distinguished in any comparison with cast oilcake films.

Canola-meal films containing cellulose nanocrystals and glycidyl methacrylate showed improvements in matrix compactness and mechanical or thermal responses [21]. Yet the reinforcing particles and reactive modifier were introduced together; the improvement cannot be attributed exclusively to the recovered fiber fraction unless the individual contributions are separated experimentally. This limits direct comparison with untreated whole meal or cake.

These findings indicate a practical comparison hierarchy: first compare different reinforcement loadings within a fixed matrix and manufacturing procedure; then compare whole meal with fiber-enriched fractions obtained from the same feedstock; finally assess whether the additional separation or compatibilization steps provide a reproducible benefit [21,29]. Studies that modify filler loading, surface chemistry and processing simultaneously identify promising composite formulations but do not isolate the mechanism of improved performance.

### 4.4. Relationships Between Protein and Polysaccharide Structure and Material Properties

The reviewed studies identify at least three different sources of a strength–barrier compromise. In isolate films, glycerol content governs plasticization, and increasing protein concentration may improve strength yet increase WVP [9]. In whole sunflower-oilcake/alginate composites, higher mixed-filler loading progressively decreases tensile strength while its influence on WVP is non-monotonic [11]. In modified canola-isolate films, SDS and stearic acid produce contrasting strength and liquid-water-uptake responses [10]. Reinforced canola-meal films add a further confounder where particle incorporation and chemical treatment are combined [21,30]. These results should be compared as within-study contrasts, not pooled into a numerical ranking. Any stronger cross-study synthesis requires documented film thickness, humidity gradient, sample conditioning, casting or thermo-mechanical conditions, testing standards and consistent units; water uptake, contact angle and WVP must remain distinct outcomes.

Comparison of individual studies reveals why nominal improvement in network cohesion cannot be taken as evidence of simultaneous improvement in barrier performance. In canola protein isolate films, Chang and Nickerson [9] found that increasing glycerol reduced tensile strength and elastic modulus while increasing elongation and water vapor permeability. Increasing the protein concentration increased strength but also increased water vapor permeability, illustrating that a mechanically stronger protein network need not provide greater resistance to moisture transport. A related, but chemically distinct, trade-off was reported by Alves et al. [23]: adding locust bean gum to sunflower protein films increased tensile strength and reduced elongation, whereas water vapor permeability remained higher than in the protein-only control. Interpretation of this comparison requires particular care because the protein-only and blended formulations also differed in glycerol content [23]. The results therefore support coupled effects of protein–polysaccharide interactions, plasticization, and water sorption, rather than establishing that the added polysaccharide alone caused the change in permeability.

### 4.5. Structural Function in Multicomponent Systems

The practical choice between whole cake or meal and isolated fractions must follow the required structural function rather than an assumed ranking of raw materials. Whole-stream formulations may be attractive where modest strength is sufficient and reduced fractionation is valued, but heterogeneous composition and limited control of filler dispersion can make repeatability difficult [11,12]. Isolate-based formulations permit targeted adjustments to film extensibility or strength [9,10] and may be more compatible with some controlled thermo-mechanical routes. For example, Rouilly et al. [24] demonstrated extrusion of sunflower-protein-isolate film and reported tensile strength of 3.2 MPa and elongation at break of 73% for an optimized processing composition; the result demonstrates process feasibility but not a mechanical advantage over whole oilcake. The food-storage result for rapeseed-oilcake packages [12] should likewise not be treated as proof of manufacturing scalability. To resolve relative performance and processing requirements, a matched study should compare (i) whole cake, (ii) defatted meal, and (iii) a protein concentrate/isolate from the same lot, using specified dry solids, plasticizer basis and either common casting or validated processing-specific controls. Film thickness, microstructure, strength, elongation, WVP, moisture-conditioning response, food-level protection and material/energy inputs should then be reported together. Until such experiments are available, claimed superiority and sustainability benefits remain conditional.

## 5. Residual Oils and Fractions Recovered from Soapstock as Hydrophobic Components

### 5.1. Chemical Characteristics of Lipid Fractions

For packaging design, the decisive difference among recovered lipid fractions is not total lipid content alone but lipid-class distribution and processing history. Residual oils in mechanically pressed cake are generally triacylglycerol-rich, whereas crude soapstock contains fatty-acid soaps, free fatty acids, acylglycerols, phospholipids, water and unsaponifiable constituents [6,7,8]. Acidulation changes this balance by converting soaps into free fatty acids and separating an acid-oil phase [6,7,22]. Even at the same nominal lipid loading, these fractions need not exhibit equivalent water interactions, dispersion, physical state or chemical stability. Accordingly, any structure–property comparison should specify neutral-lipid/free-fatty-acid/soap content, fatty-acid profile, phospholipid content, residual water and initial oxidative state rather than reporting only total extractable lipids.

Fatty-acid composition may alter melting or crystallization behavior, oxidation sensitivity and domain organization, whereas the presence of ionizable free fatty acids or soaps can modify interfacial interactions with hydrophilic polymers. However, unsaturation alone does not predict the moisture barrier of an actual composite: the final response also depends on lipid quantity, the matrix and the continuity of the lipid-rich region [3,6,7,8]. The evidence reviewed below therefore separates (i) chemical characterization of recovered fractions, (ii) within-study film measurements and (iii) moisture protection measured on food. Direct head-to-head tests of chemically defined fractions obtained from one industrial soapstock, with constant matrix and total lipid mass, remain scarce; a general ranking of triacylglycerol-rich oils versus free-fatty-acid-rich fractions is not experimentally justified.

### 5.2. Soapstock as a Source of Functional Lipid Components

The distinction between feasibility and functional superiority is particularly important for soapstock. Kuk and Ballew [13] characterized cottonseed and safflower soapstocks and produced a film from freeze-dried material after rehydration and casting. This showed that a film-forming system can be recovered without isolating a purified single lipid class. It did not, however, establish how controlled changes in free-fatty-acid, soap or phospholipid concentration affect WVP, tensile strength or structural aging in modern protein–polysaccharide composites. Nor can those properties be inferred from the mere formation of a cohesive surface layer.

Recovery conditions further complicate direct comparisons. Acidulation alters soap content and the composition of the separated acid oil; Astrausks et al. [22] varied acid identity, pH and temperature for rapeseed soapstock and reported a purified acid-oil yield of 34.7% under their optimal condition (concentrated sulfuric acid, pH 5, starting material at approximately 20 °C). This is quantitative evidence for fraction-recovery efficiency, not for film barrier performance, mechanical strength or food-contact suitability. It also shows why two fractions identified as “acid oil” need not be equivalent inputs unless their recovery histories and final compositions are characterized [6,7,8,22].

Direct food-level evidence is provided by Beaulieu et al. [14], who compared cottonseed- and safflower-soapstock-derived coatings on green bell peppers under short-term ambient storage. Both recovered streams contained approximately 55–60% free fatty acids in the reported chemical characterization, yet their moisture-retention performance differed [14]. This contrast indicates that overall free-fatty-acid percentage is an insufficient predictor: fatty-acid distribution, minor polar constituents, coating viscosity and applied layer thickness may all contribute. The study reported food weight loss, not a standardized water vapor permeability value for a self-supporting film, so the two types of endpoint must not be treated as interchangeable.

Taken together, the earlier soapstock film study [13] and the pepper-coating experiment [14] support process feasibility and a defined food-protection benefit, respectively, but neither establishes a composition–WVP–tensile-strength relationship under controlled, matched conditions. Furthermore, a more viscous coating may give stronger moisture retention but be less suitable for reproducible application. The original pepper-coating authors also identified the need for additional purification and food-grade assessment, including concerns associated with cottonseed-derived material [14]. Consequently, the potential practical benefit of avoiding lipid purification must be balanced against variation in source composition, handling, quality assurance and food-contact risk.

### 5.3. Hydrophobic Modification and Structural Continuity

A controlled model study illustrates why lipid concentration cannot be evaluated independently of the matrix. Vieira et al. [15] cast chitosan and alginate emulsion films with olive oil at 25, 50 or 100% (*w*/*w*, relative to biopolymer) and soybean lecithin at 5, 10 or 25% (*w*/*w*, relative to oil). Across the screened formulations, WVP was 1.35–2.72 × 10^−12^ mol m m^−2^ s^−1^ Pa^−1^ for chitosan and 1.70–4.73 × 10^−12^ mol m m^−2^ s^−1^ Pa^−1^ for alginate films, with measurements made under a defined humidity gradient [15]. For the chitosan films, formulations containing 25% oil with either 10% or 25% lecithin exhibited the lowest reported WVP values, whereas the alginate minimum was associated with 100% oil and 25% lecithin. The chitosan response therefore does not identify 25% lecithin as a uniquely optimal dose. Increasing oil above 25% did not monotonically improve chitosan-film WVP. The authors associated this pattern with possible droplet coalescence, but they did not experimentally isolate droplet size as the sole cause. These results are relevant mechanistic comparators, not tests of heterogeneous industrial soapstock fractions.

The same study also demonstrates that a moisture-barrier improvement need not imply weaker mechanics. In the selected chitosan and alginate formulations, tensile strength, elongation at break and Young’s modulus were not significantly different between comparable films with and without olive oil [15]. Crosslinking, rather than olive-oil incorporation, significantly increased tensile strength and stiffness while decreasing elongation. A further approximately 30% reduction in WVP occurred after crosslinking only for the selected chitosan formulation containing 25% oil and 25% lecithin; this change cannot be attributed to the lipid concentration alone. Moisture sorption was additionally reduced at high water activity (a_w = 0.91) in selected oil-containing films [15]. The joint lesson is that WVP, equilibrium water sorption, film strength and extensibility can respond to different formulation or processing variables and must be evaluated together.

In a complementary experiment using recovered industrial fractions, Beaulieu et al. [14] reported approximately 25% loss of mass (normalized to pepper surface area) for uncoated green bell peppers after 78 h under ambient conditions, compared with approximately 5% for peppers coated with cottonseed soapstock diluted 1:4. This formulation decreased moisture loss by up to 79% relative to controls, but its viscosity made application difficult. A more workable 1:8 dilution reduced mass loss by up to 48%, whereas safflower-soapstock coatings reduced it by approximately 21–25%. The tested films also varied in wax addition (0, 5 or 10%) [14]. Hence source identity, concentration, possible solid-lipid contribution and coating application were not independently controlled. These data document practical moisture protection with a dose–process trade-off; they do not establish a numerical WVP benefit or isolated effect of saturation.

### 5.4. Oxidative Stability as a Design Constraint

A hydrophobic fraction can improve the initial moisture-related response yet introduce a separate chemical-stability constraint. Residual and soapstock-derived lipid fractions containing unsaturated acyl chains may oxidize during material drying, subsequent thermal processing or storage. Their oxygen exposure, initial peroxide/secondary-oxidation status and endogenous antioxidant contents therefore matter alongside contact angle and WVP [3,6,7,8]. The long-term barrier properties of a lipid-containing film cannot be presumed stable solely because the starting formulation has a favorable laboratory WVP.

Evidence remains particularly incomplete for refining-derived fractions in packaged-food systems. Soapstock-derived inputs can carry free fatty acids, residual soaps, phospholipids, sterols, tocopherols and pre-existing oxidation products, while fractionation may introduce additional processing residues [6,7,8,22]. Such constituents may affect odor, phase behavior, migration and food-contact suitability. The short-term moisture protection observed for soapstock-coated peppers [14] does not demonstrate the oxidative stability of the coating during prolonged storage, nor does it establish food-grade status. This distinction matters because a material with lower initial water-related transport but deteriorating lipid chemistry may fail the intended food-contact function.

The most informative next experiment would compare, at fixed polymer composition and total lipid loading, a triacylglycerol-rich fraction, a free-fatty-acid-rich recovered fraction, and mixtures with controlled residual-soap/phospholipid contents from the same feedstock lot. Processing energy, emulsion or casting conditions, dry film thickness, humidity gradient, conditioned tensile strength, WVP, domain morphology, oxidative markers and migration should be measured before and after storage. A food trial with standardized coating dose or package geometry would then determine whether an apparent laboratory barrier benefit produces a reproducible improvement in preservation. Until such matched evidence exists, assigning universal superiority to a soapstock-derived lipid class would be premature [6,7,8,13,14,15,22].

## 6. Interfacial Organization and the Role of Phospholipids

### 6.1. Phospholipids at Hydrophilic–Lipid Interfaces

Phospholipids may act as interfacial modifiers because their amphiphilic molecules can adsorb at the boundary between a hydrophilic polymer phase and a dispersed lipid phase. However, the observation of lower water vapour permeability (WVP) in a lecithin-containing composite does not, by itself, establish a phospholipid-specific mechanism. The response may also be governed by the total lipid fraction, polymer identity, plasticizer, crosslinking and drying history [3,15]. An isolated lecithin effect requires otherwise comparable formulations in which phospholipid content is varied without simultaneously changing these variables. This distinction is particularly important for oil-refining streams: commercial lecithin is not compositionally equivalent to heterogeneous degumming gums containing water, neutral lipids and other polar constituents [6,7,8].

The phospholipid headgroup distribution (e.g., phosphatidylcholine and other phospholipid classes), total phospholipid content and neutral-lipid impurities should be reported before attributing a functional effect to a recovered fraction [6,7,8]. Potential benefits of additional lecithin include improved wet-emulsion dispersion; possible limitations include more hydrophilic or mobile material at high loading, altered polymer interactions, and the presence of unbound amphiphiles. Such effects are formulation-dependent hypotheses unless shown by direct concentration series or phase-characterization measurements [15]. A more stable starting emulsion must not be treated as proof of a less permeable or mechanically stronger dry film.

### 6.2. Interfacial Organization During Film Formation

Processing conditions are a separate explanatory variable in phospholipid-containing composites. In the controlled model study of Vieira et al. [15], polymer–oil–lecithin mixtures were homogenized at 13,500 rpm for 2 min, cast and dried at 22 °C for 72 h; the resulting films were subsequently subjected to a separate ionic-crosslinking step. These stages can change polymer-network mobility, phase distribution and film properties independently of nominal lecithin concentration. The authors reported WVP and tensile responses, but did not establish a single causal pathway from lecithin dose through measured droplet size to the final dry-film barrier. Their proposed interaction between lecithin choline groups and tripolyphosphate in the chitosan film is therefore a plausible interpretation, not direct molecular proof [15].

**Figure 2:**
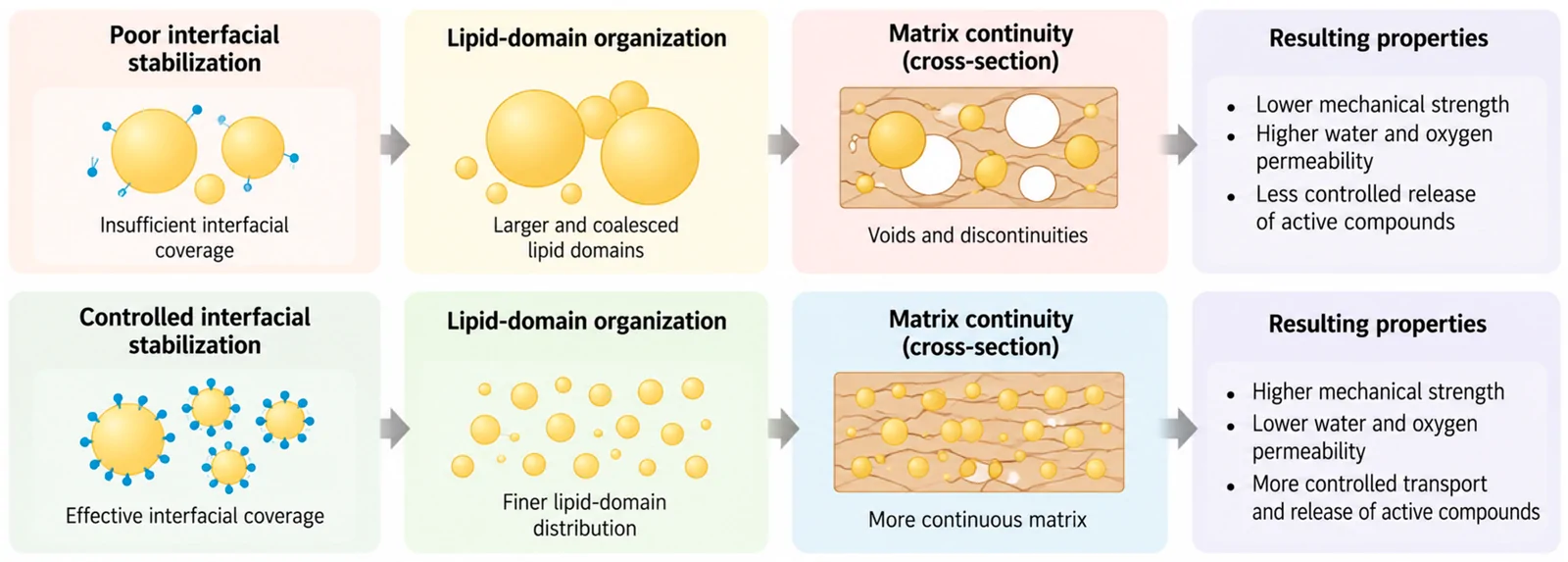
Conceptual representation of how interfacial stabilization may influence lipid-domain organization, matrix continuity, and the resulting properties of multicomponent packaging materials.

The conceptual alternatives for interfacial organization and their anticipated consequences for matrix continuity are illustrated schematically in Figure 2; they should not be interpreted as direct experimental images or universally established outcomes.

A separate non-oilseed model by Pérez-Gago and Krochta [19] found that the effect of wax particle size (approximately 0.5–2.0 µm) on WVP and mechanical properties depended on wax loading: the response was clearer at 60% than at 20%. This supports a lipid-loading-by-morphology interaction, but does not test phospholipids and cannot establish their independent stabilizing contribution. Together, these examples justify assessing droplet-size distribution before drying and domain morphology in the final material, alongside casting conditions, film thickness, WVP and mechanical performance, rather than inferring dry-film performance from emulsification alone [15,19]. Figure 2 presents this mechanism as a conceptual model requiring experimental validation for each recovered fraction.

### 6.3. Lecithin as an Interfacial Design Variable

The clearest concentration-based packaging evidence in the reviewed literature is provided by Vieira et al. [15]. Their chitosan and alginate films contained soybean lecithin at 5, 10 or 25% (*w*/*w*, relative to olive-oil mass), combined with olive oil at 25, 50 or 100% (*w*/*w*, relative to biopolymer mass). For chitosan films, low WVP values were reported at 25% oil with either 10% or 25% lecithin, while the lowest alginate-film WVP was obtained at 100% oil and 25% lecithin. The reported WVP ranges were 1.35–2.72 × 10^−12^ and 1.70–4.73 × 10^−12^ mol m m^−2^ s^−1^ Pa^−1^ for the chitosan and alginate series, respectively [15]. Thus, the favorable formulations depended on both matrix identity and lipid loading; the study did not demonstrate one uniquely optimal lecithin concentration for all chitosan- or alginate-based films. It is not evidence for a universal optimal phospholipid ratio or for similar performance of phospholipid-rich refinery by-products.

The same experiment clarifies important confounding factors. When selected oil-containing and oil-free films were compared, tensile strength, elongation at break and Young’s modulus did not differ significantly [15]; changes in these variables were driven more strongly by post-casting crosslinking. Crosslinking also further decreased WVP by approximately 30%, but only for the selected chitosan film with 25% oil and 25% lecithin; no comparable additional benefit was established for the corresponding alginate formulation [15]. A possible role of choline groups in this effect was suggested, but not experimentally isolated. Consequently, neither the water barrier improvement nor the mechanical response should be assigned entirely to increasing lecithin concentration.

Other reports demonstrate the usefulness, but also the attribution problem, of complex lecithin-containing systems. Socha et al. [26] prepared alginate–lecithin films at a fixed 5% (*w*/*w*) lecithin concentration and introduced coffee extracts at 5% (*v*/*v*); the changes among extract-containing films cannot reveal a lecithin concentration optimum. Sun et al. [31] incorporated ZnO nanoparticles supported on lecithin-modified montmorillonite into chitosan films, so changes in antimicrobial or mechanical performance reflect the hybrid system rather than a lecithin-only contrast. In a more direct food experiment, El-Sherif et al. [32] reported an optimized chitosan/lecithin/cinnamon-essential-oil bilayer with 0.25 mg cinnamon essential oil per 100 mL nanoemulsion; it exhibited tensile strength of 45.6 MPa and WVP of 1.78 × 10^−10^ g m^−1^ s^−1^ Pa^−1^ and preserved refrigerated salmon for up to 12 days. Although this supports the effectiveness of the combined active formulation, the cinnamon oil, bilayer structure and processing preclude attribution of the observed preservation effect to lecithin alone [32].

### 6.4. From Interfacial Stability to Structure–Function Control

The available evidence supports treating phospholipid content as a formulation-dependent interfacial variable, not as a stand-alone predictor of package performance. A decisive experiment should compare a lecithin-free control with a graded phospholipid series at fixed oil mass, polymer and plasticizer contents, film-forming conditions and thickness; the same design should be applied to commercial lecithin and a chemically characterized industrial phospholipid-rich fraction. A crossed oil-by-phospholipid concentration design would test interactions directly [15]. Phosphatidylcholine and other phospholipid classes, droplet-size distributions before drying, dry-film domain morphology, WVP, oxygen permeability, strength, migration and aging should be measured on the same specimens. An application-level storage test would then be required to establish whether any independently observed stabilization improves food protection. Without such controls, a decrease in WVP, stronger antibacterial activity or longer shelf life in a multicomponent film remains evidence for that full formulation rather than for the isolated contribution of phospholipids [15,26,31,32].

Comparison of lecithin-containing formulations illustrates a further limitation in attributing observed performance to interfacial stabilization alone. Vieira et al. [15] varied lipid and surfactant contents within chitosan- and alginate-based films, whereas studies involving lecithin-modified hybrid fillers [31] and chitosan/lecithin/essential-oil nanoemulsions assembled as bilayers [32] simultaneously introduced other functional ingredients and different material architectures. Improvements in one of these systems therefore cannot be assigned exclusively to lecithin without controls that separate surfactant concentration from changes in lipid loading, filler chemistry, or layer structure. The available evidence is consistent with a formulation-dependent interfacial role, but direct comparison of phospholipid-class composition, droplet-size distributions, dried-film morphology, and transport properties would be needed to demonstrate that the same mechanism operates in fractions recovered from different oilseed-refining streams [15,31,32].

## 7. Structure–Property Relationships in Multicomponent Packaging Materials

The four functional categories in Section 3 provide a useful starting point, but they become experimentally meaningful only when a defined structural feature is associated with a measured material response. The evidence below therefore distinguishes (i) features observed directly by microscopy or a controlled change in particle size, (ii) film properties measured under specified conditions, and (iii) mechanistic explanations that remain inferred rather than causally established. Comparisons are made primarily within individual studies. Results from whey-protein/beeswax models [19,33] illustrate specific physical mechanisms but do not directly validate composite formulations made from industrial oilseed side streams.

### 7.1. Mechanical Properties and Structural Continuity

Petraru and Amariei [11] examined alginate films containing 0–0.5 g sunflower oilcake per 1.0 g alginate with 0.5 g glycerol. Their scanning electron micrographs revealed that added oilcake increased surface roughness, whereas the film cross-sections remained dense and continuous, without visible pores or ruptures. Across the formulation series, tensile strength decreased from 27.11 to 8.66 MPa. The same specimens showed a non-monotonic WVP response: 1.66 × 10^−4^ in the control, 1.98 × 10^−4^ at 0.1 g oilcake, and 1.13 × 10^−4^ g mm m^−2^ h^−1^ kPa^−1^ at the highest loading [11]. Thus, greater observed surface roughness and lower strength coexisted with an ultimately improved moisture barrier, even though the imaged cross-sections did not show macroscopic structural discontinuities. SEM images alone cannot quantify sub-resolution transport paths or establish whether altered polymer–particle adhesion, lipid distribution, thickness or another factor caused the response. Attributing the strength decrease to pores or cracks would therefore go beyond the observations.

A different intervention produces a distinct mechanical response in a more chemically defined material. In solution-cast canola protein-isolate films, increasing glycerol promoted elongation while decreasing tensile strength and stiffness [9]. In formulations modified with SDS or stearic acid, the effects on mechanical behavior and water uptake diverged [10]. These results are consistent with changes in network mobility or component interactions, but they are not directly comparable to the structural changes observed in oilcake-filled alginate because the matrices, modifiers and characterization methods differ. Across studies, relevant reporting conditions include specimen geometry, conditioning humidity, tensile-test rate, film thickness and whether internal morphology was actually measured [9,10,11]. The principal finding is therefore not a universal relationship between visual smoothness and strength, but the need to measure internal continuity and mechanical performance independently.

### 7.2. Barrier and Surface Properties

The most informative morphology–barrier evidence is concentration- and processing-specific. In a controlled whey-protein/beeswax emulsion model, Pérez-Gago and Krochta [19] varied average wax-particle size from approximately 0.5 to 2.0 µm at 20 and 60% beeswax (dry basis). Particle size affected WVP and mechanical properties at 60% wax, but not at 20% [19]. This establishes a lipid-loading-dependent particle-size effect in that model, not a general rule for oilseed-derived emulsions. In the chitosan/alginate–olive-oil–lecithin study [15], the oil/lecithin combinations associated with minimum WVP differed between matrices (25% oil with 10% or 25% lecithin for chitosan; 100% oil/25% lecithin for alginate), while post-casting crosslinking reduced WVP by approximately 30% only in the selected chitosan condition. These measured formulation effects cannot establish lipid-domain size as the independently verified cause. Likewise, in another whey-protein/lipid emulsion model, drying at 80 °C reduced WVP relative to lower drying temperatures without a significant parallel change in tensile properties [33]. The studies thus show distinct dependencies on particle size, loading, matrix identity, crosslinking and drying, rather than mutually contradictory outcomes for equivalent films.

The combined evidence also illustrates why superficially similar numerical changes may be interpreted differently. An increased contact angle does not measure through-film WVP, a smooth SEM surface does not establish gas-tight internal continuity, and a reduction in WVP after drying cannot be assigned solely to lipid redistribution without independent morphology and crystallinity data [11,15,19,33]. Cross-study comparisons should report whether permeability includes thickness normalization and the applied water-activity gradient, as well as polymer/plasticizer ratios and film conditioning. Where these parameters are not harmonized, the defensible conclusion is a within-study trend and a clearly delimited mechanistic hypothesis, not a pooled numerical performance ranking.

### 7.3. Active Functionality and Material Stability

Active functionality provides an additional example of property responses that should not be conflated with a measured molecular or morphological mechanism. Tymczewska et al. [17] incorporated aqueous or methanol–water rapeseed-meal extracts at 4, 8 and 12% into gelatin-based films. Radical-scavenging activity generally increased with extract inclusion, but the measured mechanical response was not a monotonic dose–response relationship. For the 4% aqueous-extract formulation, Young’s modulus was 394 ± 32 MPa compared with 245 ± 32 MPa for the unfortified control; higher extract levels did not yield a correspondingly proportional increase [17]. Extract concentration is not equivalent to the analytically determined concentration of identified phenolic compounds, and the observed changes do not demonstrate a specific matrix–phenolic bonding mechanism. Furthermore, improved film-level scavenging is not proof of slower oxidation in packaged food. These findings are best interpreted as linked but distinct chemical-activity, mechanical and optical responses to a complex extract.

The subsequent study by Tymczewska et al. [18] examined gelatin–rapeseed-meal packaging in an accelerated 7- and 14-day rapeseed-storage experiment and reported improved antioxidant characteristics of stored rapeseed and the subsequently pressed oil. This constitutes more direct food-level evidence than the film assays in [17], but the different formulations, endpoints and accelerated conditions do not support a causal ranking of the two studies or extrapolation to commercial shelf life. Similarly, the performance of multicomponent lecithin-containing systems [26,31,32] cannot be ascribed to a single active or interfacial fraction without controlled comparators. A more decisive structure–property assessment would measure, on matched specimens, quantitative phase/domain dimensions, thickness, permeability under the same humidity gradient, tensile properties after identical conditioning, and any active-compound concentration, mobility and post-storage retention. Food-level testing is then needed to determine whether a material-level improvement leads to a practical protective effect [11,15,17,18,19,33].

### 7.4. Critical Comparison of Evidence and Limits of Transferability

The studies reviewed above provide different levels of evidence and should not be treated as directly interchangeable. Controlled protein-film experiments establish within-formulation responses to plasticizer or reinforcement [9,23,28,30], while studies using whole oil cakes or meals include additional, incompletely controlled effects of residual oil, fiber, phenolic compounds, and particle morphology [11,21]. In contrast, the rapeseed oil-cake packaging study by Vera et al. [12] went beyond material characterization by measuring oxidative deterioration of sunflower oil during storage. This product-level result is especially relevant to packaging functionality, but lower oxidation in a packaged food cannot, by itself, distinguish the contributions of oxygen-barrier properties, light attenuation, endogenous antioxidants, or other package-related factors. Inferences about specific causal mechanisms therefore require experiments that relate chemical composition and microstructure to both material transport properties and food-level endpoints within the same experimental design.

A more discriminating evidence base would compare a whole side stream, its isolated structural fraction, and a recombined multicomponent material under matched conditions. The minimum useful dataset should include the botanical and processing origin of each fraction; protein, fiber, residual-lipid, and phospholipid composition; lipid oxidation status; film thickness and conditioning relative humidity; tensile strength and elongation; water vapor and oxygen transport where relevant; and direct observations of domain size, phase continuity, and interfacial defects. Factorial studies that vary lipid loading, phospholipid content, and processing conditions independently would help separate chemical effects from morphological effects. Until such comparisons are available, claims of synergistic performance should be restricted to formulations in which the improvement exceeds that of appropriately matched single-fraction and physical-mixture controls, rather than inferred solely from the presence of chemically complementary constituents.

## 8. Processing Strategies for Films, Coatings, and Multilayer Systems

Processing determines whether the candidate functions identified in Section 3 can be realized in a finished material. Laboratory casting emphasizes formulation and drying history, surface coating adds substrate adhesion and deposited-layer continuity, and extrusion imposes shear and heating [1,3,20]. The relevant comparison is not simply whether each method produces a film, but whether a common feedstock retains mechanical integrity, barrier performance and any active constituents when processed by different routes. Processing is a condition of function, not an additional functional class.

### 8.1. Casting and Drying as Structure-Forming Processes

Solution casting allows defined formulations to be compared with relatively mild mixing, but drying can reshape the dispersed phase after an apparently stable emulsion has formed [3,15]. The evidence should be interpreted by property rather than as an undifferentiated “processing effect”. In whey-protein/lipid emulsion films, Pérez-Gago and Krochta [33] found significantly lower WVP at higher drying temperatures, including films dried at 80 °C compared with 25 or 40 °C (at 40% RH); mechanical properties were not significantly changed by the drying conditions investigated. The authors proposed altered lipid crystallinity or distribution as possible explanations, but those mechanisms should not be treated as uniquely demonstrated. This non-oilseed model shows that drying may alter transport without a parallel mechanical response. It does not establish the same effect for liquid oilcake residues or recovered soapstock fractions, whose oxidation and phase mobility must be tested separately [6,7,8,33].

### 8.2. Coatings as Functional Surface Layers

Coatings offer an alternative strategy when the recovered fraction is intended primarily to modify surface, barrier, or active properties rather than to provide the complete mechanical structure of the package. This approach can be particularly advantageous for lipid-containing formulations because hydrophobic or active functionality can be localized at the food or substrate interface without requiring the coating itself to behave as a self-supporting film [1,3]. The feasibility of using oilseed-refining side streams in this form has been demonstrated by soapstock lipid coatings applied to green bell peppers, where reduced moisture loss during storage was observed [14]. This example illustrates an important distinction between film-forming ability and packaging function: a material does not necessarily need to form an independent film to provide an effective surface-protection function. For multicomponent coatings, performance depends on wetting, adhesion, layer continuity, penetration into the substrate, and resistance to cracking or delamination. Local defects may dominate mass transport even when average coating thickness is sufficient [1,3]. On porous substrates such as paper or fiber-based materials, coating–substrate interactions should therefore be considered part of the final material architecture rather than as a secondary processing issue.

### 8.3. Extrusion and Thermoplastic Processing

Extrusion is a scalable process but imposes temperature, moisture, shear and residence-time conditions that differ fundamentally from laboratory casting [20]. For sunflower protein isolate, Rouilly et al. [24] varied die temperature (85–160 °C), water and glycerol levels and showed that regular film formation and mechanical behavior depended strongly on these variables. At high die temperature with 70 parts glycerol and 20 parts water per 100 parts isolate, the reported film reached a tensile strength of 3.2 MPa, a Young modulus of 17.7 MPa and a strain at break of 73% [24]. These results demonstrate the feasibility and formulation sensitivity of extrusion for a purified sunflower protein, not extrusion readiness of whole oilcake or a cake–soapstock–lecithin blend. In addition, high processing temperatures may change protein organization and oxidize unsaturated recovered lipids. Direct comparisons therefore require the same feedstock and chemical specification, monitored process history, and equivalent performance measurements before cast-film trends are extrapolated to industrial technologies [1,20,24].

### 8.4. Multilayer and Hybrid Structures

Multilayer architectures provide a means of spatially separating functions that may be difficult to optimize simultaneously within a single homogeneous material. Individual layers may be designed primarily for mechanical support, water or oxygen control, interfacial compatibility, or active functionality [1,3]. This strategy is particularly relevant to oilseed-derived materials because structural and hydrophobic fractions may exhibit limited compatibility when forced into a single phase. Spatial separation can therefore reduce the need to maximize all properties within one formulation. Biswas et al. demonstrated this principle by combining poly(lactic acid) with a cottonseed protein-containing layer to obtain bilayer films with measurable mechanical, thermal, optical, and water vapor transport properties [25]. For systems combining oil-cake-derived structural fractions with lipid fractions recovered from soapstock, multilayer design may offer a particularly attractive route because structural and hydrophobic functions can be assigned to different regions of the material. The design challenge then shifts from bulk miscibility toward control of interlayer adhesion, thickness uniformity, defect formation, and transport across interfaces [1,3,25].

### 8.5. Processing as a Determinant of Final Function

The existing processing studies define separate, not interchangeable evidence tiers: casting of defined isolates and cake composites [9,10,11], drying-dependent barrier performance in a non-oilseed emulsion model [33], food coating with recovered soapstock [14], and extrusion of sunflower protein isolate [24]. No study among these establishes that one common oilcake–soapstock–phospholipid formulation retains equivalent performance across all routes. A decisive experiment would apply matched composition and conditioning to at least two processing methods, quantify resulting phase organization and oxidation state, and measure mechanical, water-vapor and oxygen-barrier properties followed by a relevant food-storage endpoint. Without this design, scale-up feasibility remains distinct from demonstrated functional equivalence.

### 8.6. Application-Specific Opportunities and Limits of Current Evidence

The practical value of a recovered oilseed fraction depends on the protection task assigned to the finished package, rather than on whether the fraction can be incorporated into a film. Potential applications should therefore be distinguished by-product type, contact conditions, required structural and barrier performance, and the strength of the available evidence. In the current literature, experimental material production is considerably more common than controlled food-storage validation, and claims about application readiness need to reflect this difference [1,2,12,14,25,29].

Self-supporting films and flexible packages represent one route for protein- and polysaccharide-rich oil cakes or meals. For example, Vera et al. [12] tested rapeseed oil-cake packaging with sunflower oil and documented lower peroxide values and conjugated diene formation than in the open control after 60 days of storage. This is unusually relevant evidence because the package was evaluated with a lipid-rich food, although it does not establish industrial sealability, migration suitability, or performance across different products and storage conditions. Cast canola-protein films and extruded sunflower-protein films provide complementary information on film formation and mechanical behavior [9,10,24], but these material-scale experiments should not be interpreted as equivalent evidence of shelf-life extension. Flexible packages intended for oxygen-sensitive foods require adequate sealing, puncture resistance, and oxygen and light protection in addition to acceptable tensile properties.

Rigid or semi-rigid trays, inserts, and disposable food-service articles provide a different design route because particle reinforcement and compressive strength may be more important than high elongation at break. Rani et al. [29] prepared hot-compression-molded plates from mustard, flaxseed, and soybean meals combined with plant fibers and reported their physical and mechanical characteristics, liquid-contact response, and degradation under the investigated conditions. These findings support the technical feasibility of forming three-dimensional articles, not their general suitability for direct food contact, repeated use, hot filling, or storage of high-moisture foods. The priority tests depend on the proposed use and should include load resistance, dimensional stability, short-term wetting, extractables and migration, and validation under realistic food-contact temperatures.

Surface coatings offer a distinct opportunity for lipid-rich refining fractions when reducing moisture loss is the primary objective. Beaulieu et al. [14] applied cottonseed- and safflower-soapstock-derived lipid films to green bell peppers and observed reduced weight loss relative to uncoated controls, with the outcome depending on the source and formulation of the coating. This finding demonstrates an application-specific moisture-retention effect, but not universal performance on other fruits or vegetables, and the authors also identified a need for further cleanup and safety assessment before food-grade use. The same recovered fractions could be investigated as water-resistant surface layers on paper or fiber-based substrates; however, coating adhesion, cracking, grease resistance, and transmission of gases would need independent verification, rather than being inferred from improved wettability [3,13,14].

Multilayer structures are potentially useful when the hydrophilic structural fraction cannot independently supply the moisture resistance required by the target food. Biswas et al. [25] prepared bilayer materials comprising poly(lactic acid) and cottonseed meal and measured changes in water vapor transmission and mechanical and optical properties. These results support the architectural feasibility of combining a protein-rich layer with a more hydrophobic polymer layer, but do not demonstrate that a recovered soapstock lipid fraction or lecithin can substitute for the polymer without compromising interlayer adhesion. Likewise, the proposed combination of oil-cake structural phases, soapstock-derived hydrophobic fractions, and phospholipids is a testable formulation strategy rather than an established commercial material [3,6,7,8,15,25].

The application comparison in Table 2 identifies the level of evidence and the measurements needed to advance each route. For lipid-rich foods, proposed active liners or antioxidant-containing coatings should be prioritized only when their chemical function is linked to reduced oxidation of the packaged food and acceptable migration; the presence of tocopherols or phenolic compounds is insufficient on its own [12,16,17,18,26,27,34,35]. For moisture-sensitive products, performance should instead be judged against water transfer, surface wetting, and physical integrity in the intended humidity range. The most useful research direction is thus not to maximize the number of proposed packaging formats, but to select a limited number of food–material combinations with measurable protection needs, feasible processing, appropriate safety assessment, and demonstrable added value relative to existing packaging.

## 9. Functional Validation, Safety, Stability, and End-of-Life Performance

### 9.1. From Material Properties to Food Protection

Material screening and demonstrated food protection represent distinct levels of evidence. An increased contact angle, lower water vapor permeability (WVP), or higher DPPH/ABTS radical scavenging value does not by itself demonstrate a longer shelf life. The sunflower-oilcake/alginate film study [11] used a 10-day, 50 °C laboratory sunflower-oil oxidation challenge and measured peroxide value (PV), which is an informative product-contact model but not an oxygen transmission measurement or a commercial storage trial. More direct food-level evaluations include sunflower oil in rapeseed-oilcake packaging for 60 days at 23 °C [12], bell peppers with soapstock-derived coatings for 78 h [14], and rapeseed stored in meal-containing gelatin bags for 7 or 14 days under accelerated conditions [18]. These experiments differ in food matrix, test geometry, temperature, duration and controls, and therefore support application-specific, rather than universal, preservation claims. Film-level antioxidant or antimicrobial responses should be reported as screening endpoints unless linked to independent changes in an appropriately controlled packaged food [17,26,27].

For active materials, the dose of added material must be differentiated from the concentrations of analytically identified molecules and from the final activity of the film. Tymczewska et al. [17] prepared gelatin films containing 4, 8 and 12% water or methanol–water rapeseed-meal extracts. Direct Quencher - DPPH and Quencher - ABTS assays generally indicated higher scavenging with extract incorporation, but antibacterial effects varied among the five tested organisms and were not a monotonic function of extract dose; the authors also identified the chemical characterization of individual extract components as a subject for future work. Consequently, these 4–12% values quantify the incorporated extract, not the concentration of sinapic acid, sinapine or any other identified compound in the finished film. Reporting an assumed compound-specific dose or treating laboratory antibacterial screening as proof of preservation would overstate the study [17].

A more recent rapeseed-derived multicomponent example also illustrates both the strength and the limits of dose–response evidence. Zhuo et al. [27] reported a total phenolic content of 18.17 mg gallic acid equivalents (GAE) per g of dry rapeseed-cake extract and incorporated 5% (*v*/*v*) extract stock together with 5% (*v*/*v*) silver-nanoparticle dispersion into a chitosan-based casting formulation. For the rapeseed-cake composite (CBR-Ag), inhibition reached 43.62 ± 0.81% against Escherichia coli and 38.25 ± 4.92% against Staphylococcus aureus in the reported co-culture assay. These are measurements of the combined extract–chitosan–silver system; silver nanoparticles prevent attribution of the antimicrobial response to rapeseed phenolics alone. Total phenolics expressed in GAE are not quantitative concentrations of individually identified compounds, and the nominal extract-solution percentage cannot be converted to an individual-phenolic loading per mass of dry film without additional compositional data [27].

### 9.2. Oxidative Stability of the Packaging Material

The chemical stability of the packaging material itself represents a second level of validation, particularly when residual oils or fractions recovered from soapstock are incorporated into the formulation. Such fractions may contain unsaturated fatty acids, free fatty acids, phospholipids, tocopherols, sterols, and pre-existing oxidation products, all of which may influence stability during processing and storage [6,7,8]. Oxidation within the packaging matrix may modify both composition and structure. Changes in lipid oxidation state can affect phase behavior, molecular mobility, sensory characteristics, and potentially mechanical or barrier properties. Oxidation products formed within the material may also modify the chemical environment at the food–package interface [3,6,7,8]. Relevant analytical parameters therefore include fatty acid composition, peroxide value, secondary oxidation products, tocopherol stability, and changes in sterol composition [6,7,8,16,34]. For lipid fractions recovered from soapstock, such measurements are particularly important because oxidative susceptibility depends on the composition and processing history of the recovered fraction [6,7,8]. The presence of endogenous antioxidants should not be interpreted automatically as evidence of chemical stability. Tocopherols, phenolic compounds, and other minor constituents may provide antioxidant activity, but their effectiveness depends on concentration, accessibility, interactions with the surrounding matrix, oxygen exposure, and processing history [8,17,18,26,27]. A lipid-containing material should therefore be evaluated simultaneously for functional benefit and susceptibility to oxidative degradation.

Evidence for material stability during the intended storage period remains substantially weaker than initial film-characterization evidence. For unsaturated residual oils, acid oils and phospholipid-rich fractions, a useful paired test would determine lipid class and fatty acid composition, initial and aged PV, secondary oxidation products, tocopherol retention and potential odor-active volatiles, while re-measuring tensile and barrier properties after storage [6,7,8,16,34]. None of the cited food-protection experiments establishes that a finished cake–soapstock–lecithin composite maintains both phase integrity and oxidative stability over commercial storage. This is a defined limitation, not an inference that such composites are intrinsically unstable.

### 9.3. Migration and Food-Contact Safety

Food-contact suitability cannot be inferred from renewable, bio-based, or biodegradable origin. Materials derived from heterogeneous side streams may contain low-molecular-weight lipids, free fatty acids, phenolic compounds, residual processing components, oxidation products, and other intentionally or non-intentionally present substances that may migrate into food [6,7,8,35]. Recent analyses of bio-based and biodegradable food-contact materials emphasize that migration assessment should consider not only intentionally incorporated additives but also impurities, oligomers, degradation products, and other non-intentionally added substances. Analytical workflows increasingly combine targeted and non-targeted chromatographic approaches, particularly GC- or LC-based methods coupled with mass spectrometry [35]. Migration is influenced by both material and food characteristics, including migrant polarity and molecular size, matrix composition, temperature, contact time, surface-to-volume ratio, and food lipid content [35,36]. This is especially relevant for packaging intended for lipid-rich foods because hydrophobic food matrices may promote the transfer of lipophilic constituents. Primary studies on biodegradable packaging have shown that migration profiles can differ substantially between food simulants and real foods and may depend strongly on temperature and material composition [36]. Accordingly, overall migration alone may be insufficient when complex side-stream-derived materials are used. Specific or non-targeted analysis may be required when chemically heterogeneous fractions or degradation products are present [35,36]. Food-contact assessment should therefore be integrated into material development from an early stage rather than performed only after functional optimization. A formulation that exhibits favorable mechanical, barrier, or active properties cannot be considered suitable for food packaging unless the identity and migration behavior of relevant constituents are also adequately characterized [35,36].

The regulatory decision must be distinguished from a general demonstration of biodegradability or from a favorable result in a migration screening test. In the European Union, food-contact materials are subject to the safety principles of Regulation (EC) No 1935/2004 and the applicable good-manufacturing-practice requirements of Regulation (EC) No 2023/2006 [37,38]. The rules for plastic materials and articles in Regulation (EU) No 10/2011, as amended inter alia by Commission Regulation (EU) 2025/351, apply to the materials and layers falling within their defined scope [39,40]. Neither those plastics-specific compositional restrictions nor their migration limits should automatically be treated as universal acceptance criteria for every protein-, polysaccharide-, or lipid-based biodegradable material. The relevant rules, including any measure applicable to active or multilayer packaging, depend on the intended article, its constituent layers, food-contact conditions, and jurisdiction. Development should document the raw-material provenance, refining and fractionation aids, relevant impurities and non-intentionally added substances, and the risk assessment of potential migrants where needed [35].

The available packaging-performance studies do not constitute complete food-contact safety assessments. For example, Zhuo et al. [27] reported no detectable silver signal by energy-dispersive X-ray spectroscopy (EDS) in sampled tomato tissues, but EDS is not a sufficiently sensitive, species-specific measure of Ag or Ag^+^ migration to establish compliance with food-contact requirements. Quantitative, validated migration testing remains necessary for silver-containing films and should be complemented by assessment of extract constituents, non-intentionally added substances, oxidative degradation products and relevant food simulants or real foods. Similarly, in vitro non-cytotoxicity results for alginate–lecithin films with coffee extracts [26] do not replace food-contact migration testing and are evidence from a non-oilseed model. For heterogeneous oilseed fractions, time, temperature, surface-to-volume ratio and the lipid character of the contacting food must be specified [12,14,18,26,27,35,36].

### 9.4. Functional Validation in Lipid-Rich Foods

Evidence from actual food-contact trials is promising but uneven in relevance and duration. Vera et al. [12] compared sunflower oil stored at 23 °C for 60 days in rapeseed-oilcake packaging with EVOH packaging and an exposed control; PV and conjugated dienes were lower in both packaged groups than in the exposed oil, but the responsible oilcake constituents were not isolated. Soapstock-derived cottonseed coatings reduced bell-pepper weight loss after 78 h from approximately 25% in controls to 5% under the most protective 1:4 dilution, whereas a more practical 1:8 dilution gave a smaller protective effect [14]. Rapeseed meal/gelatin bags improved antioxidant characteristics of seeds and pressed oil after 7 and 14 days of accelerated storage [18], but the result does not independently determine an isolated extract concentration–response relationship. In an additional fresh-produce study [27], cherry tomatoes coated with the rapeseed-cake/chitosan/silver composite retained 83.6% of initial weight after 12 days at 25 °C and 50% RH, compared with 68.2% for uncoated controls. These reported findings support a food-level benefit for the tested multicomponent formulation, not protection attributable solely to rapeseed extract. However, the source publication describes an 8-day weight-loss monitoring protocol in its Methods while reporting a 12-day endpoint in its Results; the observation period therefore requires clarification. Quantitative silver-migration assessment also remains necessary.

Taken together, these studies substantiate specific food-level endpoints—oxidative markers in stored oil and seed [12,18], moisture loss in coated peppers [14], and mass retention and quality indicators in tomatoes [27]—but do not establish equivalence to commercially used packaging across products, shelf lives or environmental conditions. Stronger evidence would require paired measurements of film permeability and aging, analytically characterized active-component concentrations, released or transferred substances, and food-quality trajectories under matched packaging and storage conditions. The time-dependent retention of a food-quality parameter should not be used to infer the release mechanism of a particular active compound unless transfer has been measured.

### 9.5. Linking Safety, Stability, and Packaging Function

The proposed evidence hierarchy therefore distinguishes: (i) documented feedstock and film formulation, including the loading of whole side stream, extract, lipid and emulsifier; (ii) chemical analysis of identified active compounds, reported in a defined mass or volume basis where available, separately from total phenolics or extract percentage; (iii) a film-level antioxidant/antimicrobial result, reported with the assay and control; (iv) time-resolved food-level protection under an appropriate unprotected and conventional-packaging control; (v) chemical and mechanical stability during storage; and (vi) food-contact migration and end-of-life tests appropriate to the intended claim. Table 3 illustrates this distinction for extract-loaded gelatin films [17], a rapeseed-cake/chitosan/silver active composite [27], and an alginate–lecithin/coffee model where individual chlorogenic acids were quantified in the starting extract [26]. The latter is included only as a methodological comparison and does not directly validate oilseed side-stream formulations. Absent data are identified rather than estimated. These requirements prevent performance in a screening assay from being conflated with a food-preservation, safety or biodegradation claim [11,12,14,17,18,26,27,35,36,41].

### 9.6. Biodegradability and End-of-Life Validation

The use of renewable or food-industry-derived feedstocks does not by itself demonstrate biodegradability. Bio-based origin, biodegradability, and compostability describe different material attributes and should not be used interchangeably [5,41]. This distinction is particularly important for multicomponent materials, where crosslinking, lipid incorporation, reinforcement, or multilayer assembly may alter degradation behavior relative to the individual starting components. Biodegradation claims should therefore be supported by standardized testing under clearly defined environmental conditions. For packaging intended for industrial composting, EN 13432 specifies requirements and evaluation criteria related to biodegradability, disintegration, effects on the biological treatment process, and compost quality [42]. Complementary standardized methods, such as ISO 14855-1, determine ultimate aerobic biodegradability under controlled composting conditions by measuring evolved carbon dioxide and the degree of disintegration [43]. Simple mass loss or visible fragmentation alone is insufficient to demonstrate complete biodegradation because these measurements do not necessarily distinguish physical disintegration from biological mineralization. For packaging derived from oilseed-processing side streams, end-of-life behavior should be evaluated together with material functionality. Increasing hydrophobicity, crosslink density, or structural reinforcement may improve barrier and mechanical properties while simultaneously slowing water uptake and microbial degradation. Conversely, rapid biodegradation may be incompatible with the moisture resistance and shelf-life performance required during use [5,41]. Future material development should therefore treat biodegradability as an experimentally validated property rather than an assumed consequence of renewable feedstock origin. The relevant objective is not maximum degradation rate, but a controlled balance between stability during food storage and predictable degradation within the intended end-of-life environment (Table 4) [5,41,42,43].

**Table 3:** Recommended framework for functional validation of biodegradable packaging materials intended for lipid-rich foods.

Packaging Attribute	Material-Level Characterization	Food-Level Validation	Interpretation and Literature
Oxygen barrier	Oxygen transmission rate or oxygen permeability	Primary and secondary oxidation products; tocopherol and sterol stability	Ability to limit oxidative deterioration [16,34,44]
Water vapor barrier	WVTR or water vapor permeability	Moisture loss, mass loss, water activity where relevant	Ability to control moisture transfer [3,14]
Surface hydrophobicity	Contact angle; surface free energy	Product-surface interaction where relevant	Surface interaction with water; not a substitute for barrier measurements [3,15]
Mechanical integrity	Tensile strength, elastic modulus, elongation at break	Package integrity during storage and handling	Resistance to cracking, deformation, and structural failure [9,10,21,23,28,29,30]
Interfacial organization	Microscopy; lipid-domain size and distribution; phase continuity	Indirectly reflected in package stability and food protection	Relationship between phase organization and function [3,15,31,32]
Antioxidant functionality	DPPH, ABTS, or complementary screening assays	Oxidation markers; tocopherol retention; sterol stability; phytosterol oxidation products	Verification that antioxidant potential translates into food protection [17,18,26,27,32,44]
Lipid-phase stability	Peroxide value; secondary oxidation products; fatty acid profile; tocopherols and sterols	Evaluation of possible effects on packaged food	Verification that the hydrophobic phase remains chemically stable [6,7,8,16,34]
Migration and food-contact safety	Overall and specific migration; targeted and non-targeted migrant characterization	Analysis of food or appropriate food simulants after contact	Identification of potential exposure and safety concerns [35,36]
Long-term packaging performance	Barrier, mechanical, chemical, and microstructural properties after storage	Chemical stability of food throughout shelf life	Verification that initial material performance is maintained during use [12,16,34,44]
Biodegradability/end-of-life	Standardized biodegradation, mineralization, disintegration, and compostability testing	Not directly applicable to food	Verification of end-of-life claim under defined conditions [5,41]

**Table 4:** Selected within-study experimental evidence and its limits for function-oriented oilseed by-product packaging design.

System/Function	Formulation and Test Conditions	Reported Result (Within Study)	Interpretation and Evidence Limit
Sunflower oilcake/structural [11]	Alginate 1.0 g + glycerol 0.5 g; 0–0.5 g sunflower oilcake; cast films. SEM surface/cross-section; WVP under a 0/75% RH gradient.	SEM: increasing surface roughness but continuous, pore-free cross-sections at image resolution. TS 27.11 → 8.66 MPa; WVP 1.66 → 1.98 (0.1 g) → 1.13 × 10^−4^ g mm m^−2^ h^−1^ kPa^−1^ (maximum loading).	Morphology and properties measured in the same series: roughness did not predict the direction of WVP. No evidence that pores or cracks caused the lower TS; SEM is not proof of the molecular mechanism.
Canola isolate/structural [9]	Cast protein isolate films; protein 5.0 or 7.5%; glycerol 30–50% (protein basis)	More glycerol: strength and stiffness decrease; extensibility rises.	Single-variable responses in a more defined formulation; no matched same-lot whole-cake comparison.
Canola protein isolate/modifier trade-off [10]	Isolate-based films, solution-cast with glycerol; SDS or stearic acid as formulation modifiers	SDS improved mechanical behavior but increased water uptake; stearic acid reduced water uptake but weakened films.	Distinct mechanical–sorption trade-off within a defined isolate matrix; water uptake does not quantify WVP and this is not a whole-cake comparator.
Sunflower protein isolate/extrusion [24]	Extruded protein-isolate film; die temperature 85–160 °C; glycerol and water optimized	For a reported optimized formulation: tensile strength 3.2 MPa; elongation at break 73%.	Shows a thermo-mechanical route beyond casting, but is not a matched whole-cake/isolate comparison; swelling and operating inputs remain relevant.
Model lipid + lecithin/interfacial [15]	Cast chitosan/alginate; soybean lecithin 5/10/25% (oil mass basis), olive oil 25/50/100% (polymer mass basis). Homogenized 13,500 rpm/2 min; dried 22 °C/72 h; optional post-casting crosslinking.	WVP chitosan 1.35–2.72 × 10^−12^, alginate 1.70–4.73 × 10^−12^ mol m m^−2^ s^−1^ Pa^−1^; low chitosan WVP at 25% oil with 10% or 25% lecithin; alginate minimum at 100% oil/25% lecithin. Crosslinking reduced WVP by ~30% only for selected chitosan 25%/25% film.	Matrix, oil loading and crosslinking confound phospholipid-specific interpretation. No universal optimum, direct measured headgroup-mediated crosslinking or industrial-gum validation.
Alginate/lecithin + coffee extract/fixed-phospholipid model [26]	Alginate 2.5% (*w*/*v*) + fixed lecithin 5% (as formulated) and 5% (*v*/*v*) green/light/medium/dark coffee-bean extract; solution-cast model, not an oilseed-processing fraction.	Identified extract 5-caffeoylquinic acid (5-CQA): 2052.66 mg/L in green and 1350.17 mg/L in light-roasted coffee; film TPC up to ≈275 µg GAE/g and measurable DPPH activity [26].	Quantified individual compounds in feed extract, but not their amounts retained in finished film. Coffee is a non-oilseed comparator; no food-storage or regulatory migration trial; fixed lecithin level prevents dose optimization.
Chitosan/lecithin/essential-oil bilayer/food protection [32]	Ultrasound-assisted C/L/CEO nanoemulsion top layer; optimized cinnamon oil 0.25 mg per 100 mL nanoemulsion; refrigerated salmon test.	Optimized film: TS 45.6 MPa; WVP 1.78 × 10^−10^ g m^−1^ s^−1^ Pa^−1^; salmon preservation up to 12 days.	Food-level outcome supports the combined bilayer + essential-oil formulation, not a lecithin-only causal effect. Study system and WVP units differ from [15].
Whey protein + beeswax/model morphology [19]	Whey-protein isolate/beeswax emulsion film; measured mean wax-particle size ≈0.5–2.0 µm; 20% vs. 60% wax (dry basis).	Particle size affected WVP and mechanical properties at 60% beeswax, but not at 20% [19].	Controlled concentration × particle-size interaction in a non-oilseed model. No direct evidence for complex recovered lipid/phospholipid fractions.
Whey-protein/lipid emulsion film; drying history [33]	Laboratory-cast non-oilseed emulsion films, drying at 25, 40 or 80 °C; barrier compared at defined RH.	Higher drying temperature reduced WVP (notably 80 °C vs. lower temperatures); tested mechanical properties did not change significantly.	Processing-dependent barrier change without parallel tensile change; lipid-organization explanation not independently established for recovered oilseed lipids.
Rapeseed meal extracts/active [17]	Gelatin films cast with 4, 8 or 12% aqueous or methanol–water rapeseed-meal extract [17]; laboratory film-level antioxidant and bacterial assays (five strains).	Increasing extract inclusion generally improved QUENCHER-DPPH/ABTS scavenging; antibacterial responses were organism- and formulation-dependent, with no uniform dose response. 4% aqueous extract: Young’s modulus 394 ± 32 vs. 245 ± 32 MPa control.	Reported % is extract inclusion, not identified sinapic acid/sinapine content in finished film; activity not a direct food-storage outcome, and extract-alone causal contribution is not fully resolved.
Rapeseed-cake extract + silver nanoparticles/active and food validation [27]	Chitosan film: 5% (*v*/*v*) rapeseed-cake extract stock and 5% (*v*/*v*) AgNP dispersion; extract TPC 18.17 mg GAE/g dry extract; fresh tomato 25 °C, 50% RH, 12 days.	CBR-Ag: microbial inhibition *E. coli* 43.62 ± 0.81%, *S. aureus* 38.25 ± 4.92% (12 h co-culture); tomatoes retained 83.6% vs. 68.2% mass in uncoated control after 12 d.	Food-level benefit proven for combined chitosan/extract/AgNP material, not extract alone. Total phenolics ≠ specific analyte dose; EDS-negative silver signal ≠ validated migration study; soil burial ≠ standardized mineralization. The source Methods specify 8-day tomato weight monitoring, whereas the Results present a 12-day endpoint.
Rapeseed oilcake/food protection [12]	Rapeseed oilcake packages vs. EVOH and exposed controls; sunflower oil, 23 °C, 60 days	Film TS 1.18 MPa; PV and conjugated dienes lower in packaged groups than exposed control.	Direct food test; constituents not isolated and commercial-scale performance untested.
Cottonseed/safflower soapstock/film formation [13]	Industrial soapstock; freeze-drying, pulverization, rehydration and casting	Rehydrated fractions formed a gel that could be cast and dried into a coherent thin film.	Evidence of film preparation only; no matched lipid-class loading versus WVP/tensile-property series.
Rapeseed soapstock/fraction processing [22]	Acidulation screen using selected acids at pH 2–7 and 20/45/70 °C; acid-oil purification	Purified acid-oil yield 34.7% for concentrated H_2_SO_4_, pH 5 and ≈20 °C starting soapstock.	Evidence for recovery-route dependence only; does not measure packaging WVP, mechanics, stability or food-contact suitability.
Soapstock-derived coating/food protection [14]	Cottonseed/safflower soapstock on bell peppers; 21–24 °C, 60–75% RH, 78 h comparison; dilution 1:4 or 1:8; wax 0/5/10%	Weight loss ≈25% control vs. ≈5% with cottonseed 1:4 (up to 79% reduction); practical 1:8 reduced loss up to 48%; safflower reduced loss ≈21–25%.	Direct food-level moisture retention, not measured free-film WVP. Source chemistry, viscosity, dosing and coating thickness were not separately isolated; food-grade evaluation remains necessary.
Rapeseed meal/food protection [18]	Gelatin–rapeseed-meal bags; rapeseed stored 7 and 14 days under accelerated conditions	Improved antioxidant characteristics of rapeseed and cold-pressed oil under accelerated 7- and 14-day storage; no independent analyte-specific dose–response or full commercial shelf-life evidence.	Real food-level oxidation-related endpoint for whole meal in gelatin, distinct from film-only extract study [17]; different formulation and assay prevent pooling, and migration/standardized end-of-life validation remains incomplete.

Notes: TS, tensile strength; WVP, water vapor permeability; SEM, scanning electron microscopy; RH, relative humidity; SDS, sodium dodecyl sulfate; TPC, total phenolic content; GAE, gallic acid equivalents; PV, peroxide value; AgNP, silver nanoparticles; EDS, energy-dispersive X-ray spectroscopy; CEO, cinnamon essential oil. Concentrations and permeability units are reproduced as reported by the cited studies; values obtained under different conditions or using different units should not be ranked directly.

A specific example illustrates why environmental claims must be bounded by the actual test. Zhuo et al. [27] reported apparent disappearance of chitosan/rapeseed-extract/silver-composite film samples during approximately three weeks of soil burial (with around 30% loss after one week and approximately 70% after two weeks). These observations support material disintegration under the tested conditions but do not demonstrate complete mineralization, characterize persistent silver residues, or establish industrial compostability. Soil burial, apparent mass loss and visual fragmentation should therefore be distinguished from the standardized carbon-dioxide evolution, disintegration and compost-quality assessments required by relevant standards [42,43]. The combined food-level and end-of-life data in [27] are informative, but they do not eliminate the need for migration and ecotoxicity testing of nanoparticle-containing materials.

The distinction between measurements of the packaging material and direct indicators of food preservation is summarized in Table 3, together with the corresponding functional interpretation and representative supporting studies.

Table 3 presents an evaluation framework linking measurements of packaging materials to endpoints relevant to food preservation; it does not establish that a particular formulation protects food. A comparison of experimental evidence requires information on the actual component loadings, material preparation, film thickness, characterization conditions and the measured food response, where available. Accordingly, Table 4 synthesizes selected within-study findings and distinguishes experimentally observed outcomes from mechanistic interpretations, while indicating why numerical rankings across dissimilar formulations or test methods would be inappropriate.

### 9.7. A Function-Oriented Validation Sequence for Lipid-Rich Foods

To operationalize the proposed function-oriented framework, development should begin with a defined food-protection demand rather than with the maximum feasible incorporation of a side stream. For a specified food and shelf life, the first step is to identify the dominant failure pathway (for example, oxygen- and light-driven lipid oxidation or moisture loss), the relevant exposure conditions, and a conventional package used as a reference. Candidate fractions are then selected for explicitly assigned structural, hydrophobic, interfacial, or active functions and screened for compositional variability and pre-existing oxidation. Processing conditions should be chosen to preserve those functions, followed by paired measurements of microstructure, mechanical and barrier properties, stability on aging, and migration. The final decision requires direct evidence of food protection under the intended storage conditions, together with food-contact suitability and validated end-of-life performance [1,3,12,16,35,36,41].

This sequence also establishes decision points at which a formulation should be rejected or redesigned. An apparently improved water barrier would not justify a candidate that exhibits increased oxygen permeability when oxygen is the principal cause of food deterioration. Similarly, an antioxidant-containing coating that lowers peroxide formation in a short assay should not be considered successful if the effect disappears during prolonged storage, if the coating loses integrity, or if relevant migrants cannot be shown to be acceptably controlled. Because the evidence base is uneven, the same level of certainty should not be assigned to established oil-cake food-protection experiments [12], model interfacial-film studies [15,19,33], and hypothetical combinations involving recovered soapstock and lecithin. The proposed framework is therefore a testable design-and-validation sequence, not an assertion that every oilseed by-product has demonstrated packaging suitability.

## 10. Research Gaps and Future Design Priorities

### 10.1. From Side-Stream Identity to Compositional Specifications

The unresolved comparison identified in Section 4 is whether compositional control gained by fractionation produces a reproducible functional advantage over using a whole oilseed-processing stream. In alginate films, increasing sunflower oilcake caused a substantial reduction in tensile strength while WVP responded non-monotonically [11]; studies of isolated canola proteins [9,10] used different formulations and testing conditions and do not provide a direct head-to-head answer. A decisive experiment should begin with one chemically characterized rapeseed (or sunflower) processing lot and prepare matched whole-cake, defatted-meal, protein-enriched and fiber-enriched formulations at controlled dry solids, plasticizer loading, film thickness and conditioning humidity. Strength, elongation, WVP, oxygen transport and microstructure should be assessed alongside fractionation yield, water/reagent use and energy inputs. Repeating this design across processing lots would determine whether improved consistency or performance is sufficient to justify fractionation [1,2,9,10,11,12].

### 10.2. Interfacial Organization as a Controllable Design Variable

Section 5, Section 6 and Section 7 reveal two distinct uncertainties that should not be conflated: the effect of lipid chemistry and loading on the final film, and the additional contribution of phospholipid-mediated interfacial organization. Soapstock-derived coatings have limited moisture loss in an actual food [14], but the chemical complexity of industrial soapstock and differences in dispersion, dilution and application conditions prevent inference that a particular lipid class or loading is optimal for a cast biopolymer film [6,7,8,14]. The first experiment should compare chemically characterized neutral-oil-rich, free-fatty-acid-rich and recovered soapstock lipid fractions within the same matrix and across matched dry-film lipid loadings. Measurements of contact angle, thickness-normalized WVP, tensile properties, phase morphology and oxidative aging would establish whether any hydrophobic gain is offset by structural defects or chemical instability [3,6,7,8,14,15].

A separate factorial design should isolate the effect of phospholipids. Vieira et al. [15] reported different oil/lecithin combinations associated with minimum WVP in chitosan and alginate films, while other lecithin-containing systems included additional active or reinforcing constituents [26,31,32]. These observations do not establish a universal lecithin optimum or prove that lecithin alone caused the reported gains. At fixed lipid class, loading, matrix chemistry and crosslinking conditions, compare a lecithin-free control with graded phospholipid additions, first using commercial lecithin and then chemically characterized industrial gums. Report phospholipid class profile, emulsion droplet size before drying, lipid-domain distribution in the dried film, WVP, oxygen transport, tensile strength and elongation. Only effects persisting in otherwise-matched controls should be attributed to the interfacial fraction; finer dispersion without a measurable packaging benefit is not, by itself, evidence of better design [15,19,26,31,32].

### 10.3. Processing Windows and Industrial Translation

The processing limitation identified in Section 8 is the scarcity of matched evidence showing whether composition–structure–property relationships survive translation beyond laboratory casting. Existing demonstrations of sunflower-protein-isolate extrusion [24] and emulsion-film drying effects [33] concern different experimental systems and cannot establish the scalable performance of whole-cake/soapstock/phospholipid composites. In addition, a processing-induced improvement in WVP need not be accompanied by better mechanics [33].

A useful comparison would process the same chemically characterized formulation through laboratory casting and a technically appropriate scale-up route (for example, film extrusion for a self-supporting material, or continuous coating for a coating formulation). Where equivalent thickness cannot be achieved, test matched application-specific basis weight as well as explicitly reporting film thickness and method limitations. The experiment should record water and solvent use, pH, solids content, shear, temperature, residence time, yield and energy demand. After processing and during storage, track film defects, phase continuity, lipid oxidation, loss or transformation of active constituents, barrier properties and mechanical integrity. Such paired trials would distinguish an intrinsic composition effect from a process-specific microstructure and identify formulations whose benefits persist under manufacturable conditions [1,20,24,25,33].

### 10.4. Integrated Validation of Performance, Safety, and End-of-Life

The food-protection evidence in Section 9 is application-specific rather than directly comparable across oil, seeds and fresh produce. Rapeseed-oilcake packaging reduced selected oxidation markers of sunflower oil [12], soapstock coatings limited pepper moisture loss [14], and rapeseed-meal films or active composites showed benefits in other food-contact trials [18,27]. However, different food matrices, exposure conditions, controls and storage periods preclude a general claim that one functional fraction or composite design is superior. Moreover, extract percentages and laboratory antioxidant assays [17] do not necessarily identify the retained concentration or causal role of individual active compounds, particularly when silver nanoparticles or other additives are co-formulated [27].

The priority experiment should therefore combine a matched packaging comparison with time-resolved food-quality measurements. For one target food, compare the proposed material, an otherwise-identical matrix without the candidate active or lipid fraction, an appropriate conventional reference and an exposed control; hold temperature, illumination, food mass, contact area and headspace constant. Where active functionality is claimed, include a graded extract-dose series and analytically quantify identified molecules in the dry material and, when relevant, their release or transfer during storage. Pair WVP/oxygen-barrier measurements and aged-film integrity with food endpoints appropriate to deterioration (e.g., PV, secondary oxidation products and tocopherol retention for edible oils, or microbiological and sensory endpoints for suitable perishables). Demonstrated protection should be defined against the matched food controls, not inferred from film-only DPPH/ABTS or contact-angle responses [12,14,16,17,18,26,27,34,44].

Finally, promising food-protection results must be evaluated alongside migration, chemical stability and end-of-life behavior. The silver-containing composite in [27] illustrates the evidence gap: a negative EDS observation on food cannot substitute for validated quantitative silver migration, and soil-burial mass loss cannot establish ultimate mineralization. For the same optimized and storage-aged material, identify plausible migrants and non-intentionally added substances, perform appropriate overall/specific migration tests, and evaluate chemical changes in recovered lipids and additives under food-contact conditions [27,35,36]. If biodegradability or compostability is claimed, use a relevant standardized mineralization method (e.g., ISO 14855-1), together with the additional disintegration and quality criteria required for compostability (EN 13432) [41,42,43].

These research gaps are interdependent, but they are not equally actionable. Feedstock specifications are needed before formulations can be compared; interfacial characterization is needed before a barrier improvement can be assigned to an individual fraction; and processing reproducibility is needed before laboratory performance can be extrapolated to manufacturing [1,3,15,20]. Only then can protection of a defined food be meaningfully tested alongside migration and end-of-life requirements [12,16,35,36,41]. A particularly informative benchmark would report, for the same formulation, the origin and composition of the recovered fractions, lipid-domain morphology, oxygen and water vapor transport, changes during aging, and oxidation outcomes in a relevant packaged food. This evidence sequence prioritizes causal understanding and application fitness over the addition of further, unvalidated material concepts (Table 5).

The principal unresolved questions and the matched experiments required to address them are mapped to explicit decision criteria in Table 5; the cited studies substantiate the identified gaps, whereas the experimental designs are recommendations of the present review.

## 11. Conclusions

Oilseed-processing side streams should not be considered merely as alternative raw materials for biodegradable packaging. Their value lies in the chemical complementarity of structural, hydrophobic, interfacial, and active fractions and in the possibility of assigning these fractions defined functions within multicomponent material systems [1,2,3,6,7,8].

The comparative evidence does not establish a universally superior fraction or formulation. Within a single alginate study, adding sunflower oilcake improved WVP at high loadings but reduced tensile strength and initially worsened WVP at low loading [11]. In model chitosan and alginate systems, the lipid/lecithin concentrations associated with minimum WVP were different and were affected by crosslinking [15]. Food-storage experiments with rapeseed-oilcake packaging, soapstock coatings, and rapeseed-meal-containing films provide more direct but application-specific evidence of protection [12,14,18]. These results support a function-oriented classification as a method for organizing comparisons; they do not yet validate the hypothesized combination of all four recovered functions within one industrially viable system.

Future progress requires matched comparisons of whole and enriched fractions, isolation of lipid and phospholipid effects, and validation under manufacturable processing conditions. Food protection, migration and standardized end-of-life behavior should be assessed together. Chemical complementarity remains a testable design strategy rather than a proven advantage: only reproducible, safe protection of a defined food can justify greater formulation or processing complexity [5,12,15,17,18,26,27,35,36,41,42,43,44].

## Figures and Tables

**Table 1:** Main oilseed-processing side streams, their dominant chemical constituents, and potential functions in biodegradable food-packaging materials.

Stream/Processing Stage	Main Constituents	Assigned Material Function	Evidence and Limitations
Oil cake—Mechanical pressing	Proteins, polysaccharides, fiber, residual oil, phenolic compounds	Structural/reinforcing; Active, lipid-related	Heterogeneity, hydrophilicity, compositional variability [1,2,11,12]
Oilseed meal—Extraction after pressing	Proteins, fiber, polysaccharides, low lipid content	Structural; Reinforcing, active	Need for processing or fractionation [1,2,17,18]
Residual oil/cake oil—Pressing/recovery from oil cake	Triacylglycerols, fatty acids, tocopherols, sterols	Hydrophobic; Plasticizing, potentially active	Susceptibility to oxidation, risk of coalescence [1,3,6,8]
Gums/phospholipid-rich fraction—Degumming	Phospholipids, neutral lipids, other polar compounds	Interfacial; Polarity modification	Compositional variability, water and impurities [6,7,8,15]
Lecithin—Purification of phospholipid-rich fraction	Phosphatidylcholine and other phospholipids	Regulation of interfacial organization; Emulsification, microstructure modification	Concentration- and formulation-dependent effects [15,26]
Soapstock—Alkaline refining	Fatty acid soaps, free fatty acids, TAG/DAG/MAG, phospholipids, water, sterols	Source of lipid fractions; Interfacial, potentially active	High heterogeneity, water content, need for standardization [6,7]
Acid oil/acidulated fraction—Soapstock acidulation	Free fatty acids, neutral lipids, unsaponifiable constituents	Hydrophobic; Plasticizing	High susceptibility to oxidation, odor, compositional variability [6,8]
Deodorizer distillate—Deodorization	Free fatty acids, sterols, tocopherols, other volatile/minor compounds	Potentially active; Source of hydrophobic constituents	Need for purification and safety assessment [6,8]

Notes: TAG, triacylglycerols; DAG, diacylglycerols; MAG, monoacylglycerols. Roles are potential and may depend on feedstock composition and processing.

**Table 2:** Application-specific packaging routes for oilseed-processing by-products: demonstrated evidence and outstanding validation requirements.

Potential Application	Fraction/Design Role	Current Level of Evidence	Priority Validation Tests
Flexible films and pouches for lipid-rich foods	Oilcake or protein-rich continuous phase; residual constituents may modify opacity and oxidation	Rapeseed-oilcake packaging tested with sunflower oil [12]; canola- and sunflower-protein films characterized mostly at the material level [9,10,24]	Oxygen and light barriers; sealability, puncture resistance and migration; direct oxidation markers and matched shelf-life comparisons
Molded trays and single-use serving articles	Mustard-, flaxseed- or soybean-meal matrices reinforced with plant fibers	Laboratory compression-molded plates with mechanical, moisture-response and degradation measurements [29]; no general food-contact approval demonstrated	Wet strength, thermal resistance, dimensional stability and intended-use food-contact exposure; relevant migration testing
Fresh-produce coatings	Cottonseed- or safflower-soapstock-derived lipid coatings on produce	Reduced moisture loss demonstrated for coated bell peppers [14]; response depended on formulation and botanical origin	Coating adhesion, uniformity, produce respiration and sensory response; chemical cleanup and food-contact suitability
Bilayers and functional surface coatings	Cottonseed meal/protein combined with PLA; lipid layers on fiber-based substrates remain proposals	Cottonseed/PLA bilayers characterized for water vapour transport and mechanics [25]; recovered-soapstock paper coating not validated by that experiment	Interlayer adhesion, defects, grease and moisture resistance, end-of-life assessment and full-article safety
Active liners and coatings for oxidation-sensitive foods	Protein-rich substrate with antioxidant-bearing fractions and optional interfacial modifiers	Food-level oxidation reduction shown for a whole rapeseed-oilcake package [12]; multicomponent controlled-release systems remain hypothetical [17,18,26,27]	Chemical dose and release, oxidative stability, secondary oxidation and sensory endpoints; migration and durable effect over shelf life

Notes: PLA, poly(lactic acid). Proposed tests are application-specific validation priorities and do not imply that a formulation has received food-contact authorization.

**Table 5:** Evidence-linked research priorities, matched comparative experiments, and decision-relevant endpoints for oilseed-derived packaging.

Evidence Gap (Identified in This Review)	Unresolved Question	Minimum Matched Experiment	Decision-Relevant Evidence
Whole cake vs. enriched fractions; evidence from non-matched systems [9,10,11,12]	Does fractionation improve performance enough to justify additional processing?	One characterized oilseed batch: whole cake, defatted meal, protein- and fiber-enriched fractions; fixed solids/plasticizer; replicated lots.	TS, elongation, WVP, oxygen barrier, morphology, film thickness, fraction yield, water/energy use and between-lot reproducibility.
Hydrophobicity vs. barrier and mechanical trade-offs; soapstock/composite evidence not directly comparable [6,7,8,14,15]	Which lipid class and dose improve bulk barrier performance without damaging the matrix or accelerating oxidation?	Same biopolymer matrix and matched lipid loads: neutral-oil-rich vs. FFA-rich vs. recovered soapstock fractions; lipid-free control.	Contact angle alongside WVP, TS/elongation, lipid-domain defects, PV/secondary oxidation markers after storage.
Lecithin benefits confounded by matrix, lipid and other constituents [15,26,31,32]	Does phospholipid addition independently improve dry-film function?	Factorial zero/graded lecithin × fixed lipid levels with constant crosslinking and processing; commercial vs. recovered phospholipids.	Phospholipid composition; droplet size and dried-domain distribution; WVP, oxygen barrier, mechanics; optional matched food trial.
Limited cast-to-process translation; model extrusion/drying evidence [24,33]	Do measured advantages survive a manufacturable route?	Same formulation and feedstock processed by casting and appropriate extrusion/coating route, with documented thickness and thermal/shear history.	Defects, phase continuity, aged-film barrier/mechanics, active-compound retention, yield and energy/solvent demand.
Film assays do not consistently predict food outcomes; active-component contribution unresolved [12,14,17,18,26,27]	Does a given formulation delay deterioration of a target food, and which fraction is responsible?	Time-resolved target-food trial: full film, matrix-only/fraction-minus control, conventional package, exposed control; analytically quantified active doses.	Food oxidation/microbial and sensory trajectory, parallel package aging and permeability, extract identity/loading, measured release.
Migration and biodegradation evidence incomplete, including Ag-containing systems [27,35,36,41,42,43]	Is the effective formulation food-contact safe and are environmental claims verified?	Same final and aged films: targeted/non-targeted migration plus standardized biodegradation/compostability tests and constituent-residue assessment.	Quantitative migrants and exposure context; ISO 14855-1 mineralization; EN 13432 disintegration/compost-quality criteria where claimed.

Notes: WVP, water vapor permeability; TS, tensile strength; FFA, free fatty acids; PV, peroxide value. The proposed comparative tests and decision criteria are author recommendations, not experiments already carried out in the cited publications.

## Data Availability

No new data were created or analyzed in this study. Data sharing is not applicable to this article.
